# Crosstalk between the tricarboxylic acid cycle and peptidoglycan synthesis in *Caulobacter crescentus* through the homeostatic control of α-ketoglutarate

**DOI:** 10.1371/journal.pgen.1006978

**Published:** 2017-08-21

**Authors:** Irnov Irnov, Zhe Wang, Nicholas D. Jannetty, Julian A. Bustamante, Kyu Y. Rhee, Christine Jacobs-Wagner

**Affiliations:** 1 Microbial Sciences Institute, Yale University, West Haven, CT, United States of America; 2 Department of Molecular, Cellular, and Developmental Biology, Yale University, New Haven, CT, United States of America; 3 Department of Microbiology and Immunology, Weill Cornell Medical College, New York, NY, United States of America; 4 Division of Infectious Diseases, Department of Medicine, Weill Cornell Medical College, New York, NY, United States of America; 5 Howard Hughes Medical Institute, Yale University, New Haven, CT, United States of America; 6 Department of Microbial Pathogenesis, Yale School of Medicine, Yale University, New Haven, CT, United States of America; Universidad de Sevilla, SPAIN

## Abstract

To achieve robust replication, bacteria must integrate cellular metabolism and cell wall growth. While these two processes have been well characterized, the nature and extent of cross-regulation between them is not well understood. Here, using classical genetics, CRISPRi, metabolomics, transcriptomics and chemical complementation approaches, we show that a loss of the master regulator Hfq in *Caulobacter crescentus* alters central metabolism and results in cell shape defects in a nutrient-dependent manner. We demonstrate that the cell morphology phenotype in the *hfq* deletion mutant is attributable to a disruption of α-ketoglutarate (KG) homeostasis. In addition to serving as a key intermediate of the tricarboxylic acid (TCA) cycle, KG is a by-product of an enzymatic reaction required for the synthesis of peptidoglycan, a major component of the bacterial cell wall. Accumulation of KG in the *hfq* deletion mutant interferes with peptidoglycan synthesis, resulting in cell morphology defects and increased susceptibility to peptidoglycan-targeting antibiotics. This work thus reveals a direct crosstalk between the TCA cycle and cell wall morphogenesis. This crosstalk highlights the importance of metabolic homeostasis in not only ensuring adequate availability of biosynthetic precursors, but also in preventing interference with cellular processes in which these intermediates arise as by-products.

## Author summary

Bacteria are well-known for their remarkable ability to multiply, a property that we often aim to control. To successfully self-replicate, bacterial cells must generate energy and building blocks through central metabolism and synthesize cell wall material to reproduce their shape and size. How cellular metabolism and cell wall growth are integrated during cellular replication remains poorly understood. In this work, we demonstrate the importance of the global regulator Hfq for maintaining the homeostasis of central metabolites in *Caulobacter crescentus*. Specifically, we show that accumulation of central metabolite α-ketoglutarate caused by the loss of Hfq inhibits an enzymatic reaction needed to produce cell wall building blocks. This metabolism-dependent perturbation of cell wall synthesis results in cell morphological defects and renders the cell more susceptible to cell wall-targeting antibiotics. Given that central metabolism and cell wall biosynthesis are broadly conserved, our findings suggest a new approach for combinatorial drug design.

## Introduction

Central metabolism is crucial for generating energy and biosynthetic precursors during cell growth and proliferation. In bacteria, cellular replication also requires the synthesis of peptidoglycan (PG), a major component of the bacterial cell wall that determines the shape and size of the cell. Both central metabolism and cell morphogenesis have been extensively studied, but often independently of each other.

A connection between these two processes is supported by the observation that many mutations that affect cell shape and size are found in metabolic genes [1–11]. One mechanism that connects central metabolism to cell morphogenesis in general and cell division in particular is through the action of ‘moonlighting’ enzymes. In addition to performing their normal metabolic functions, moonlighting enzymes indirectly promote or inhibit septal PG synthesis in a metabolite-dependent manner through their interaction with a component of the cell division apparatus [2, 4, 6–8]. A second mechanism that links central metabolism to cell morphogenesis has been suggested by genetic studies [1, 3, 12]. In this case, the proposed link is more direct as it relies on the availability of shared metabolic substrates. For instance, glycolysis and PG synthesis use common metabolites, such as fructose-6-phosphate (F6P) and phosphoenolpyruvate (PEP), as substrates [13]. Therefore, mutations in metabolic genes may alter metabolic fluxes that generate F6P and PEP, causing a depletion of these substrates and ultimately affecting PG synthesis.

Hfq is an RNA chaperone and a global regulator of gene expression that is involved in many aspects of bacterial physiology and stress response [14–17]. Deletion of *hfq* can affect the expression of up to 20% of the genes in the genome, including metabolic genes [18, 19]. Interestingly, the loss of Hfq in various bacteria results in varying degrees of cell morphological defects [20–25]. In this study, we show a critical role for Hfq in maintaining metabolic homeostasis in *Caulobacter crescentus* that reveals a previously unrecognized mechanistic link between metabolic dysregulation, PG synthesis and cell morphogenesis.

## Results

### Hfq affects growth and cell morphology

A recent genome-wide Tn-Seq study in *C*. *crescentus* annotated *hfq* (*CCNA_01819*) as essential for viability in PYE medium at 30°C [26], which is a common laboratory growth condition for *C*. *crescentus*. When we attempted to generate an *hfq* deletion by allelic gene replacement with an oxytetracycline resistance cassette (S1A Fig), we were able to obtain Δ*hfq* colonies. However, the Δ*hfq* colonies were much smaller than expected for normal growth on PYE plates at 30°C. The Δ*hfq* strain also grew considerably slower than wild-type CB15N (WT) in liquid culture (doubling time of ~250 min *vs* ~90 min), consistent with a severe loss of fitness. Whole-genome sequencing verified the *hfq* deletion and the absence of suppressive mutations (data deposited in the Sequence Read Archive database as SRP105792). The discrepancy with the Tn-Seq study regarding the essentiality of *hfq* is addressed in a later section of the manuscript.

**Fig 1:**
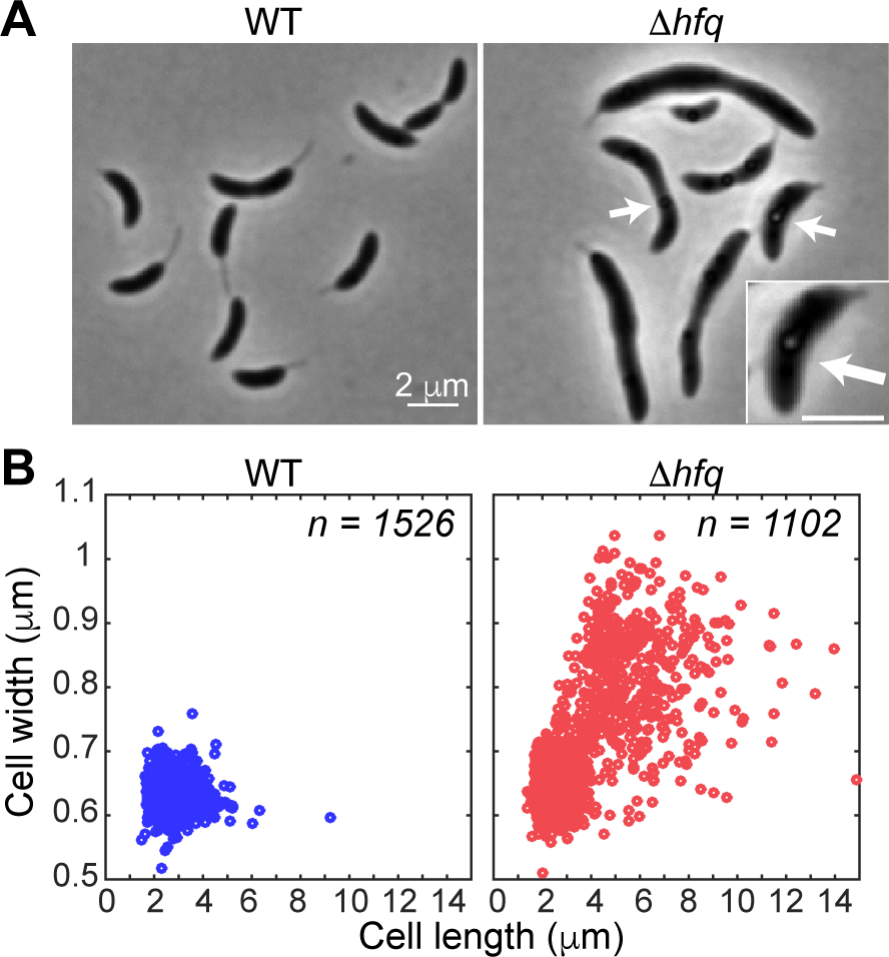
The loss of Hfq results in cell morphology defects. (A) Phase contrast images of the parental (WT) and Δ*hfq* cells taken from liquid PYE cultures grown at 30°C. The image shown for the Δ*hfq* strain highlights cells with morphology defects. Arrows denote the presence of granules in Δ*hfq* cells. Higher magnification of a representative cell containing a granule is shown in the inset (scale bar, 2 μm). (B) Scatter plots of cell lengths and widths of WT and Δ*hfq* cells grown as in (A).

Phase-contrast microscopy of Δ*hfq* cells revealed the presence of storage granules in some cells (Fig 1A, arrows), a common indicator of stressful conditions [27–30]. More interesting to us, however, was the association of the *hfq* deletion with a cell morphology phenotype (Fig 1A). While the parental CB15N strain (WT) maintained a narrow distribution of cell lengths (*l* = 2.83 ± 0.68 μm, mean ± standard deviation) and widths (*w* = 0.63 ± 0.02 μm), the Δ*hfq* strain displayed large variability in cellular dimensions (*l* = 3.99 ± 2.26 μm, *w* = 0.72 ± 0.11 μm; S1 Table) due to an abnormally high frequency of wide and elongated cells in the population (Fig 1B). In the *C*. *crescentus* genome, *hfq* is the first gene in an operon that also contains *hflX*, a gene predicted to encode a ribosome-associated GTPase (S1A Fig). A Δ*hflX* strain showed neither growth nor cell shape defects (S1B–S1D Fig), indicating that the Δ*hfq* phenotypes were caused by the loss of Hfq, and not by a polar effect on *hflX* expression.

### Suppression of Δ*hfq* phenotypes by inactivation of a metabolic gene

**Fig 2:**
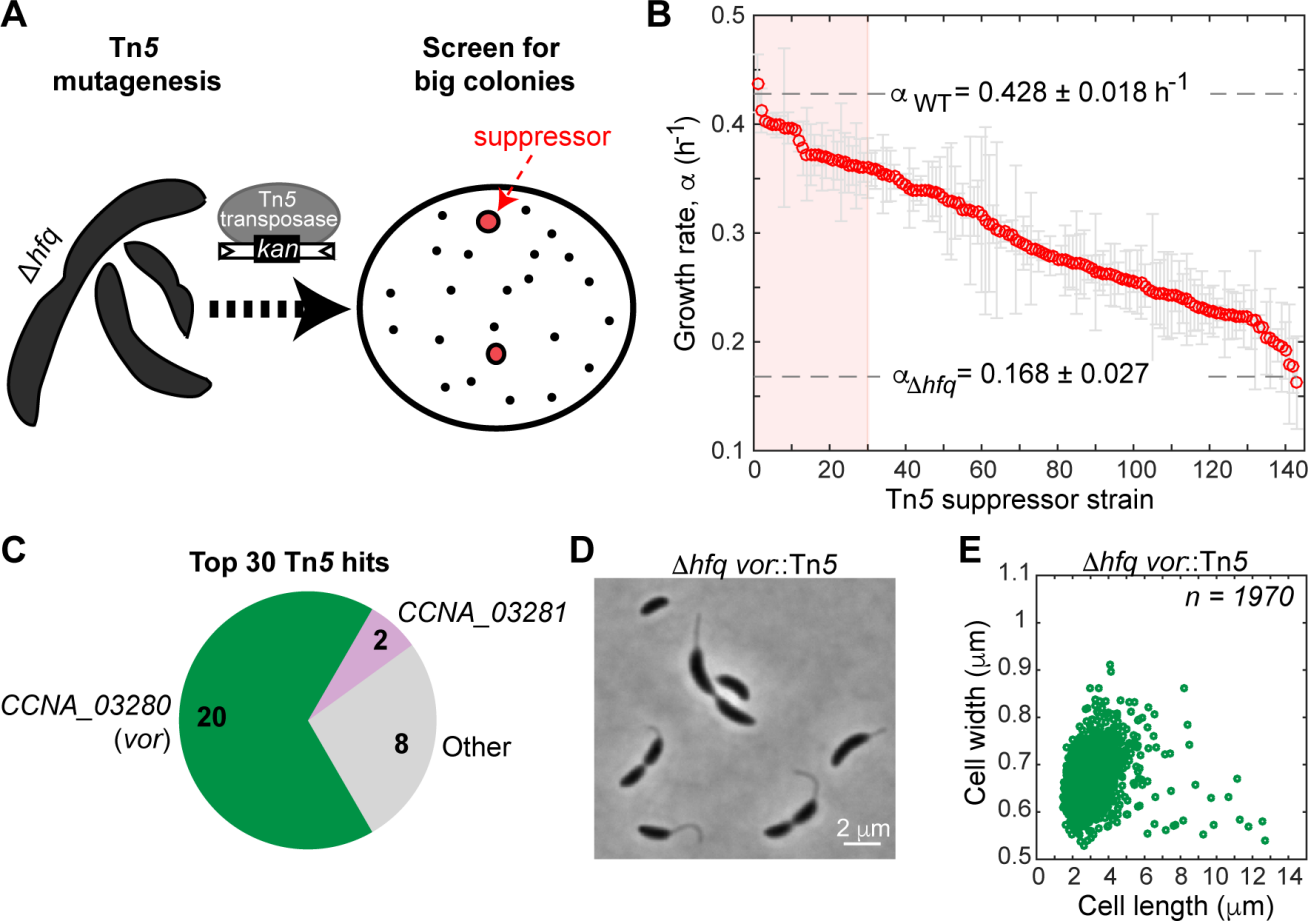
Identification of Tn*5* suppressors of the Δ*hfq* growth and cell shape phenotypes. (A) Schematic of the Tn*5* screen. The suppressors were identified based on their ability to form bigger colonies on PYE plates. (B) Growth rate measurements for Δ*hfq* suppressors grown at 30°C in 96-well plates of PYE liquid cultures. The suppressor strains are ordered from fastest to slowest growth rate. Each circle represents an average of 3 replicates with the standard deviation plotted in grey. The growth rates for the WT and Δ*hfq* strains are shown for comparison. The red shaded box represents the top 30 fastest growing suppressors. (C) Tn*5* insertion sites for the top 30 suppressors (shaded region in panel B). (D) Phase contrast image of the most common suppressor, Δ*hfq vor*::Tn*5*, grown in PYE liquid culture at 30°C. (E) Scatter plot of cell lengths and widths of the population described in (D).

To investigate the origin of the Δ*hfq* phenotypes, we first undertook a genetic approach. We took advantage of the Δ*hfq* growth defect to isolate ‘suppressor’ mutants following Tn*5* mutagenesis (Fig 2A). From ~74,000 Tn*5* mutant colonies inspected on PYE agar plates, 143 of them (~0.2%) appeared to form noticeably bigger colonies compared to the parental Δ*hfq* strain, indicating faster growth. The majority of these suppressors also showed markedly improved growth rates in liquid culture (Fig 2B).

To identify the potential mechanism of suppression, we mapped the transposon insertion sites for the top 30 fastest-growing suppressors (Fig 2C, S2 Table). This set represented suppressors with growth rates within 16% of the wild-type rate (Fig 2B, red shaded region). Two thirds (20/30) of the transposon hits mapped to a single uncharacterized gene: *CCNA_03280*, which we renamed *vor* because of its putative role in branched-chain amino acid utilization, as explained below. Another two hits were found in the adjacent gene, *CCNA_03281*, which encodes an Lrp-like transcription factor that typically senses amino acids [31]. The remainder of the Tn*5* hits were scattered around the genome (S2 Table). We focused our attention on *vor* given the prevalence of its inactivation among suppressor mutants. In addition to rescuing the growth defect in PYE medium, the *vor*::Tn*5* mutation partially suppressed the abnormal cell shape/size distribution caused by the *hfq* deletion (*l* = 2.87 ± 1.2 μm and *w* = 0.66 ± 0.05 μm; Fig 2D and 2E, S1 Table). Partial suppression of the growth and cell shape phenotypes was also observed for a Δ*hfq* strain, in which we replaced the *vor* gene by an antibiotic resistant cassette (S2A–S2D Fig, S1 Table). Inactivation of *vor* alone (either with the Tn*5* insertion or by gene deletion) in a *hfq*^*+*^ background did not show any apparent growth or morphological phenotypes (S2B, S2E and S2F Fig, S1 Table).

Based on sequence homology, *vor* is predicted to encode a 1147-residue enzyme (VOR) belonging to the 2-oxoacid:ferredoxin oxidoreductase superfamily, known to be involved in the metabolism of keto acids [32]. Recent work in *Phaeobacter inhibens*, a marine bacterium in the same α-proteobacterial class as *C*. *crescentus*, proposes that a homolog to CCNA_03280 (renamed VOR) may be involved in branched-chain amino acid (BCAA) utilization in place of the typical branched-chain keto acid dehydrogenase complex (BCKDC), which catalyzes the decarboxylation of branched-chain keto acid into acyl-CoA [33]. The *C*. *crescentus* genome appears to encode all of the necessary enzymes for BCAA degradation, except BCKDC (S3A Fig) [34]. We therefore hypothesized that VOR is the enzyme responsible for metabolizing branched-chain keto acids in *C*. *crescentus*. To test this idea, we monitored the ability of WT and *vor*::Tn*5* strains to utilize BCAAs (leucine, isoleucine and valine) as carbon sources in a defined minimal medium (M2BCAA). In agreement with our hypothesis, the *vor*::Tn*5* mutant, unlike WT, was unable to utilize the supplemented BCAAs for growth (S3B Fig), though it showed similar growth to WT in the presence of glucose as the sole carbon source (S3C Fig). Note that the small amount of growth observed for the *vor*::Tn*5* strain in M2BCAA could be attributed to the vitamin mixture included in this growth medium (S3D Fig). This vitamin mixture contains small amount of myo-inositol, a known carbon source for *C*. *crescentus* [35]. Previous microarray experiments in *C*. *crescentus* have shown that *vor* expression is induced in the amino acid-containing PYE medium relative to amino acid-free media [36]. Together, these data support a role for VOR in the BCAA degradation pathway in *C*. *crescentus*, most likely in metabolizing branched-chain keto acids, as previously suggested for *P*. *inhibens*. VOR is the name used to define the subfamily of 2-oxoacid:ferredoxin oxidoreductases involved in BCAA utilization [32, 37].

The fact that deletion of *vor* suppressed, at least partially, the Δ*hfq* phenotypes (Fig 2) indicated that *vor* expression is deleterious in the Δ*hfq* strain. To test whether the enzymatic activity of VOR was required for its observed toxicity in the Δ*hfq* background, we expressed wild-type (WT) or catalytically inactive (E84A) VOR proteins from a plasmid in the Δ*hfq* Δ*vor* double mutant background, and found that the growth and morphology defects of the Δ*hfq* strain depend on the enzymatic activity of VOR (S4 Fig, S1 Table).

### Defects in the Δ*hfq* strain are associated with perturbations of central metabolites

Since VOR is a metabolic enzyme, we considered the possibility that the Δ*hfq* defects might be caused by metabolic perturbations. We therefore conducted metabolite profiling experiments using liquid chromatography-mass spectrometry (LC-MS) to quantify the abundance of intracellular metabolites in Δ*hfq* and control strains grown in PYE. We expected that if one or more metabolites were involved in the Δ*hfq* defects, their levels would be abnormal in the *hfq* mutant and be at least partially rescued in the suppressor Δ*hfq vor*::Tn*5*. For these metabolomics experiments, we adapted a filter culture-based method [38, 39] for *C*. *crescentus* growth (S5A Fig), and verified that the defects of the Δ*hfq* strain observed in PYE liquid cultures were reproduced under these conditions (S5B Fig).

**Fig 3:**
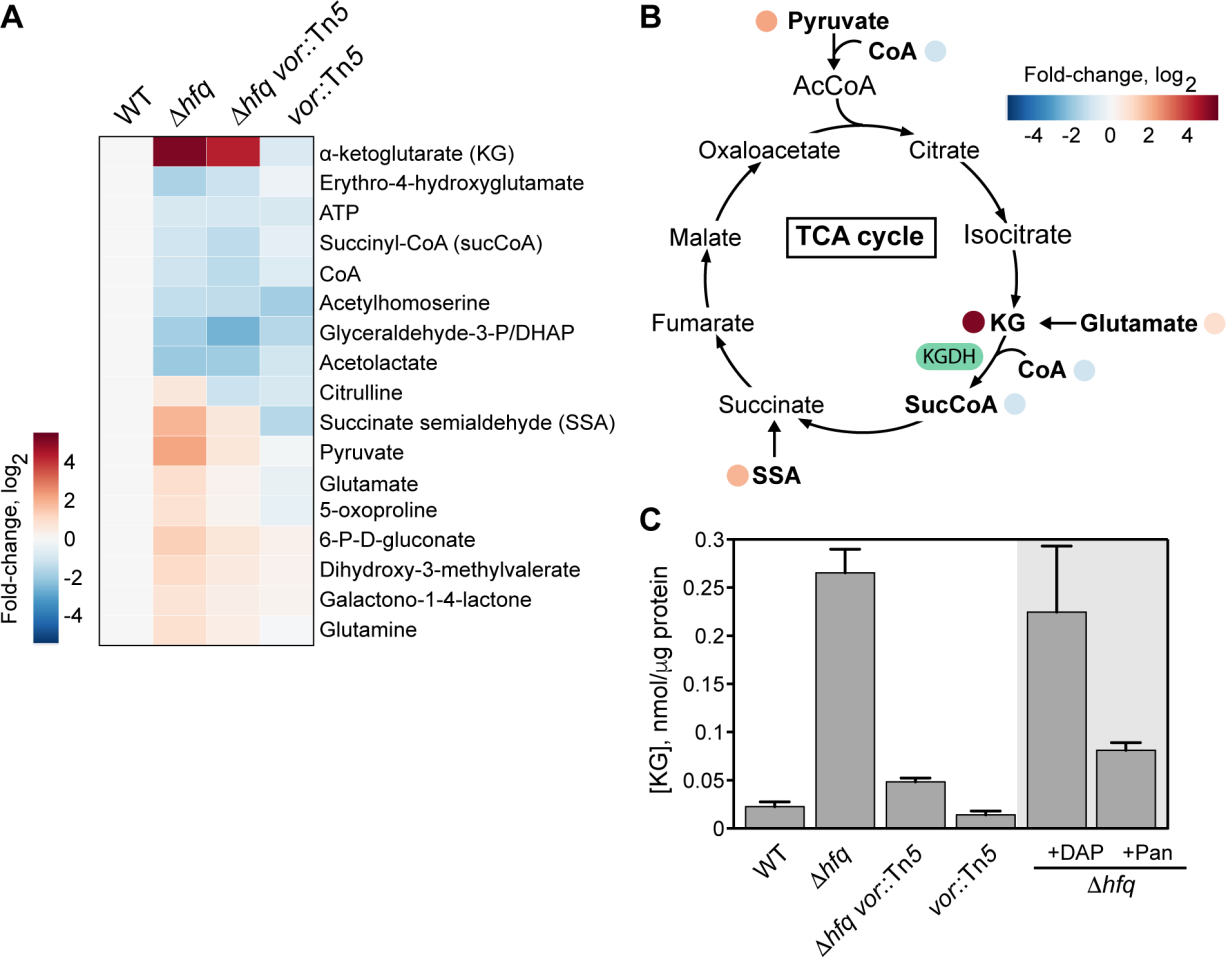
Deletion of *hfq* impacts central metabolism. (A) Heatmap showing the changes in the level of various metabolites among the WT, Δ*hfq*, Δ*hfq vor*::Tn*5*, and *vor*::Tn*5* strains, as measured by LC-MS. Cells were grown on membrane filters on top of PYE agar. Only the metabolites that were significantly increased or decreased in Δ*hfq* compared to WT (*p*-value ≤ 0.05, one-way *t*-test) are shown. Fold changes were calculated based on the mean of normalized ion counts from 3 biological replicates. (B) Perturbation in the abundance of TCA cycle metabolites between WT and Δ*hfq* cells based on data shown in (A). (C) Quantification of KG levels in various strains from cells grown in PYE liquid cultures using an enzymatic assay. KG amounts were normalized to the protein content in the metabolite extracts. Error bars denote the standard deviation from 3 biological replicates.

The metabolic profile of the Δ*hfq* strain revealed major alterations in steady-state levels of various central metabolites compared to the wild-type strain (Fig 3A, S3A Table). Many of the metabolites were closely associated with the TCA cycle (Fig 3B). Perturbations in the TCA cycle provides a potential explanation for the growth defect of the Δ*hfq* strain, as the TCA cycle is expected to play a crucial role in energy production when amino acids are the main carbon sources.

However, it was less intuitive how this metabolic dysregulation may account for the observed cell morphogenesis defects. To address this question, we focused our attention on α-ketoglutarate (KG), as this metabolite showed the most drastic change, with ~35-fold increase in the Δ*hfq* strain relative to WT based on LC-MS analysis. The abundance of KG was partially decreased in the Δ*hfq vor*::Tn*5* strain (Fig 3A), consistent with the partial suppression of the Δ*hfq* morphological defects in this strain (Fig 2D and 2E). An enzymatic assay on metabolite extracts from liquid cultures independently confirmed the correlation between the intracellular level of KG and the severity of the cell shape defects (Fig 3C), though with different fold changes that may result from differences in growth conditions (solid versus liquid media), metabolite extraction procedures [40] and quantification methods.

**Fig 4:**
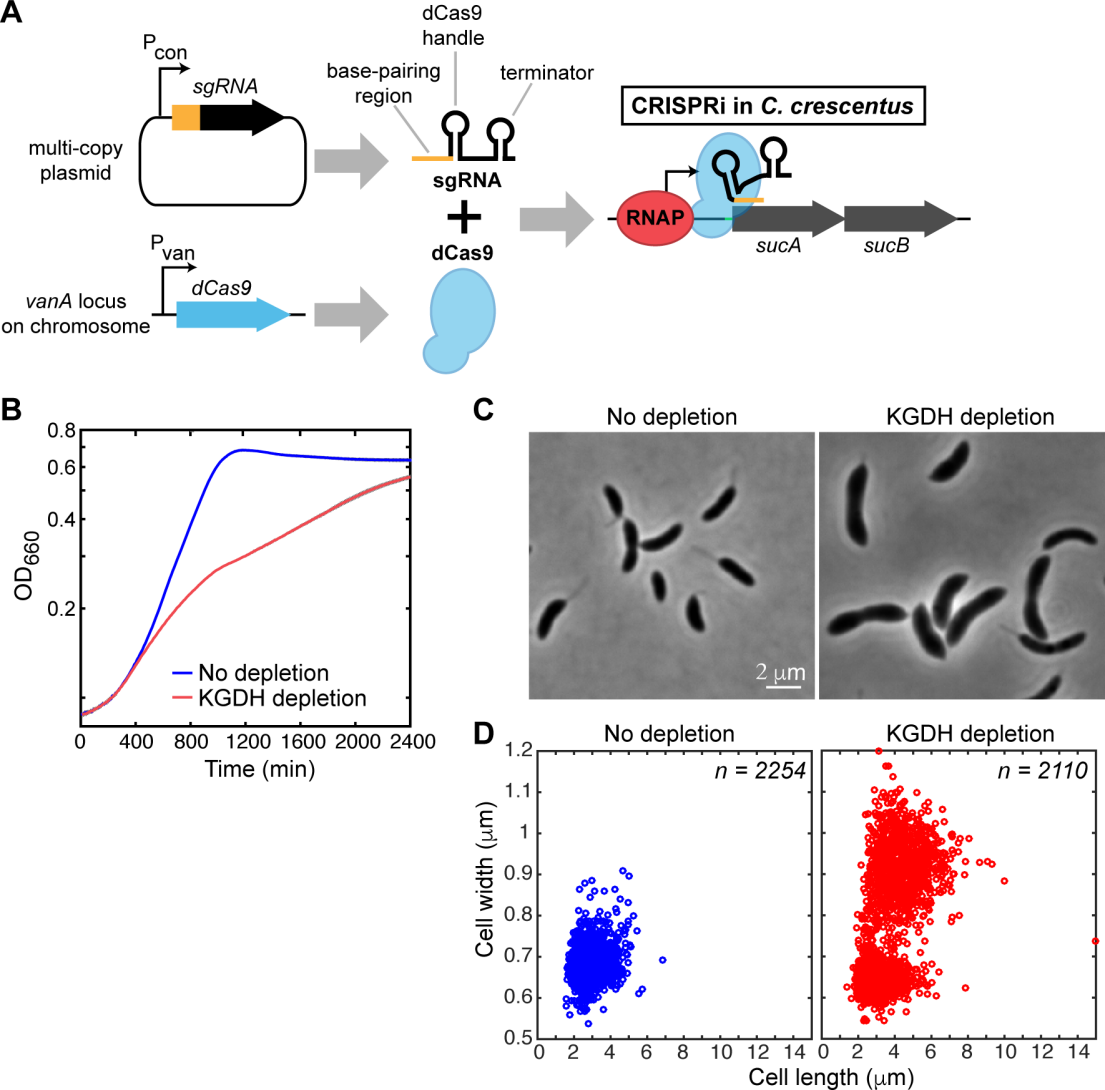
Depletion of KGDH using CRISPRi causes growth and morphology defects. (A) Schematic representation of the CRISPRi method in *C*. *crescentus*. The gene encoding dCas9 was inserted at the chromosomal *vanA* locus. sgRNA was expressed under a constitutive promoter (Pcon) from a multi-copy plasmid. To achieve KGDH depletion, the vanillic-acid-inducible dCas9 protein was directed to the *sucAB* locus by sgRNA targeting a 20-bp sequence near the *sucA* start codon resulting in transcriptional interference. RNAP stands for RNA polymerase. (B) Growth curves of the KGDH depletion strain grown at 30°C in PYE with and without 0.05 mM vanillic acid. Each curve represents the average of three replicates with the standard deviation shown in grey. (C) Phase contrast images of cells grown in the presence (KGDH depletion) or absence (no depletion) of 0.05 mM vanillic acid for 20 h in PYE at 30°C. (D) Scatter plots of cell lengths and widths of populations described in (C).

We reasoned that if KG accumulation was the cause of the Δ*hfq* morphological phenotypes, an increase in KG levels independently of an *hfq* mutation (i.e., in an *hfq*^+^ background) would phenocopy the cell shape defects. To test this prediction, we sought to increase the intracellular KG concentration by reducing the activity of the ketoglutarate dehydrogenase enzyme (KGDH), which converts KG into succinyl-CoA (SucCoA; Fig 3B). KGDH is a multisubunit enzyme complex. The E1 and E2 subunits are encoded by the operon containing *sucA* (*CCNA_00342*) and *sucB* (*CCNA_00343*) genes. Both genes are essential for viability [26]. Attempts to deplete E1 and E2 by placing the *sucAB* operon under a vanillic acid-controllable promoter (Pvan) failed, as the resulting strain did not show any growth defects when cultured in the absence of vanillic acid, presumably due to an incomplete repression of Pvan. To achieve better repression, we adapted the CRISPRi system [41] to *C*. *crescentus* (S1 Text). We validated the CRISPRi system by depleting the essential cell division protein FtsZ, which led to the expected cell filamentation phenotype (S6 Fig). To control KGDH levels, we created a CRISPRi construct targeting *sucA*. Since *sucA* and *sucB* are located in an operon, our CRISPRi construct is expected to deplete both E1 and E2 proteins (Fig 4A). Using CRISPRi, we found that KGDH depletion led to a growth defect (Fig 4B) and an increase in average cell size (Fig 4C and 4D, S1 Table), though we noted a subpopulation of cells that did not show considerable change in length and width (Fig 4D), possibly due to incomplete depletion of KGDH in these cells.

In a separate project, we isolated two independent strains carrying different point mutations in *sucA* that are temperature-sensitive (ts) for viability. These mutants exhibited normal cell shape and size at 28°C, but the cells became wider and longer when the cultures were switched to the non-permissive temperature (38°C) (S7 Fig, S1 Table). These results support the notion that KG accumulation, independently of an *hfq* mutation, is sufficient to cause cell morphological defects.

### KG accumulation causes a reduction of peptidoglycan precursor biosynthesis

**Fig 5:**
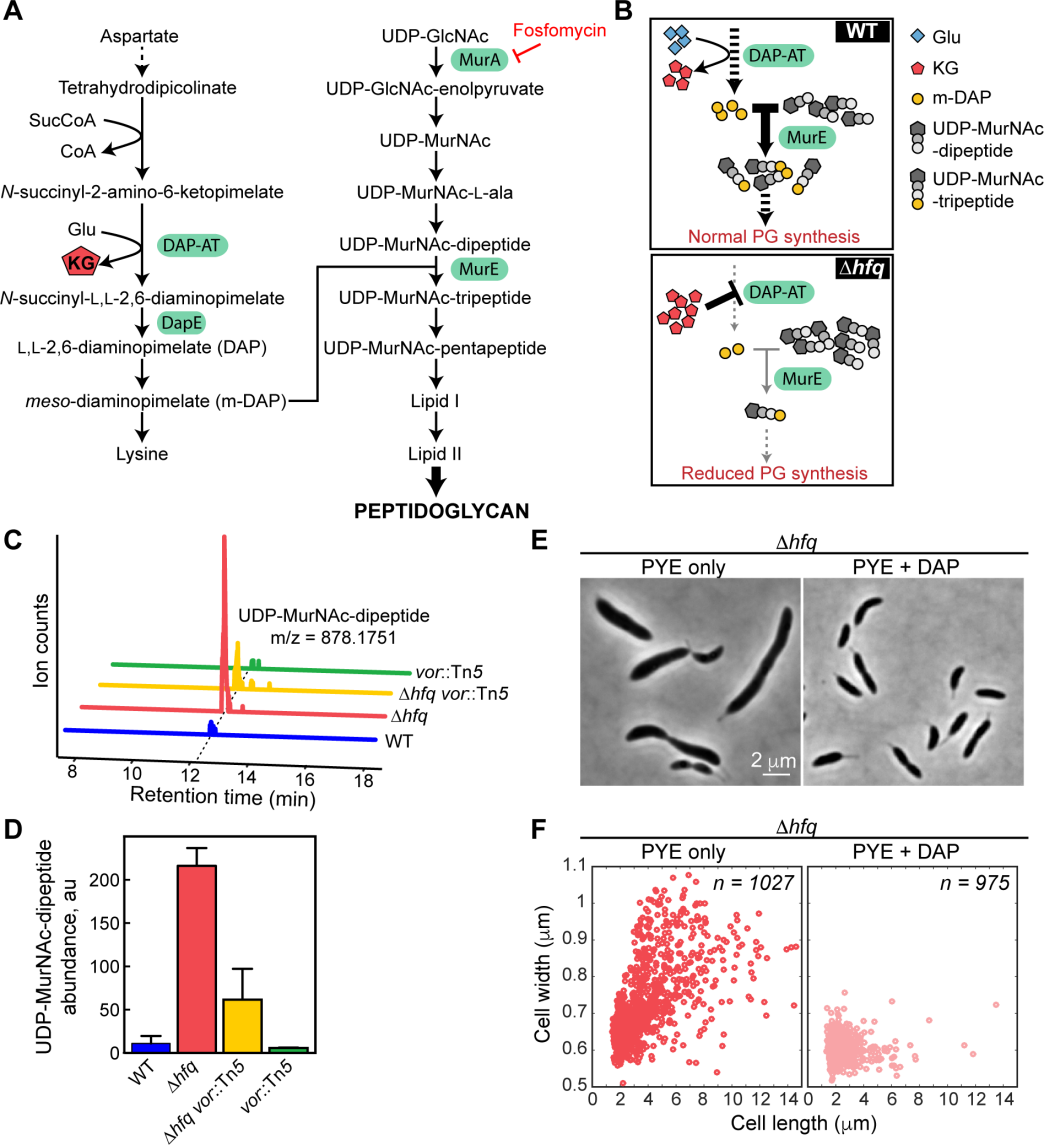
Peptidoglycan (PG) precursor synthesis is limited in the Δ*hfq* strain. (A) Schematic of the PG biosynthesis pathway in *C*. *crescentus*. The solid arrows represent a single enzymatic step, while the dashed arrows represent multiple enzymatic steps. (B) Proposed mechanism for the reduction of PG precursor synthesis in the absence of Hfq. In the Δ*hfq* strain, KG accumulation leads to the inhibition of DAP-AT activity resulting in reduced m-DAP and PG synthesis. (C) Representative LC-MS chromatograms for UDP-MurNAc-dipeptide from WT, Δ*hfq*, Δ*hfq vor*::Tn*5*, and *vor*::Tn*5* cells grown on membrane filters on top of PYE agar at 30°C. (D) Quantification of UDP-MurNAC-dipeptide level from samples described in (C) using the integrated peak intensity normalized by the protein concentration in the metabolite extracts. The error bars represent the standard deviations from 3 biological replicates. (E) Phase contrast images for Δ*hfq* cells grown in PYE in the absence and presence of 100 μM DAP for 8 h at 30°C. (F) Scatter plots of cell lengths and widths of populations described in (E).

How can KG accumulation lead to a cell shape defect? A cell widening phenotype is often characteristic of a PG defect. For instance, mutations in the cell envelope biosynthesis pathway that lead to a limitation in the lipid II PG precursor cause morphological phenotypes reminiscent of those observed in our study [42, 43]. Depletion of proteins involved in the synthesis of PG precursors has similarly been shown to increase cell width [9]. In addition, treatment with a sublethal concentration of fosfomycin, an antibiotic that inhibits an early step of PG precursor biosynthesis (Fig 5A), resulted in wider and elongated cells (S8 Fig) [44]. This phenotypic connection raised the possibility that KG accumulation in Δ*hfq* cells might affect cell morphology through inhibition of the PG precursor synthesis pathway.

Interestingly, KG is a by-product in the synthesis of *meso*-diaminopimelate (m-DAP) (Fig 5A), which is incorporated into PG precursors at the third position of the peptide side chain of PG in *C*. *crescentus* and other bacteria [45]. The enzymatic reaction producing KG is catalyzed by a succinyldiaminopimelate aminotransferase, also known as DAP-AT (Fig 5A). Over 50 years ago, biochemical work with purified DAP-AT from *E*. *coli* showed that a high level of KG inhibits the activity of this enzyme [46]. This *in vitro* observation led us to hypothesize that accumulation of KG in Δ*hfq* cells may be sufficient to inhibit DAP-AT activity and therefore m-DAP production *in vivo*. A reduction in the m-DAP pool could, in turn, limit the synthesis of PG precursors and cause cell shape defects (Fig 5B).

Our hypothesis led to two key predictions. First, inhibition of DAP-AT activity should lead to accumulation of UDP-*N*-acetylmuramoyl-l-alanyl-d-glutamate (UDP-MurNAc-dipeptide), the intermediate in the PG biosynthesis pathway immediately before m-DAP addition (Fig 5A and 5B). Therefore, we examined the Δ*hfq* metabolome for an ion matching the calculated mass of UDP-MurNAc-dipeptide (C_28_H_43_N_5_O_23_P_2_, m/z = 878.1751). As expected, we found a large peak with a retention time of ~12.22 min in the Δ*hfq* metabolome, while the corresponding peak was barely detectable in the WT sample (Fig 5C and 5D). The abundance of this ion was lower in the suppressor Δ*hfq vor*::Tn*5* strain (Fig 5C and 5D), consistent with the partial reduction in KG level in this strain (Fig 3A and 3C). To verify that this peak actually corresponded to UDP-MurNAc-dipeptide, we analyzed the chromatographic profiles of the same ion from a strain in which DapE was depleted. The *dapE* gene (*CCNA_00277*) encodes a succinyl-diaminopimelate desuccinylase, which is necessary for m*-*DAP synthesis and functions downstream of DAP-AT in the pathway (Fig 5A). Depletion of the DapE counterpart in *B*. *subtilis* results in UDP-MurNAc-dipeptide accumulation [47]. Similarly, we observed accumulation at the 12.22-min peak identified in the Δ*hfq* chromatogram when we depleted *C*. *crescentus* DapE using CRISPRi (S9A Fig), confirming that this peak corresponds to UDP-MurNAc-dipeptide. Notably, DapE depletion also resulted in the appearance of wider and more elongated cells (S9B Fig, S1 Table), consistent with the idea that m-DAP depletion causes a cell shape defect.

The second prediction of our hypothesis was that, if inhibition of DAP-AT activity and the resulting limitation of m-DAP synthesis cause the morphological defects of the Δ*hfq* strain, addition of a downstream metabolite that bypasses DAP-AT should increase m-DAP availability for PG synthesis and restore normal cell morphology. To test this prediction, we grew the Δ*hfq* strain in the presence of 2,6-L,L-diaminopimelate (DAP), which can be converted into m-DAP *in vivo* (Fig 5A). Consistent with the prediction, DAP supplementation rescued the morphology phenotypes of the Δ*hfq* strain (Fig 5E and 5F, S1 Table), despite the level of KG remaining high (Fig 3C; *p*-value = 0.38 by two tailed *t*-test, in comparison with Δ*hfq* cells grown without DAP). We noticed, however, that the Δ*hfq* growth defect was not suppressed by DAP (S10 Fig), suggesting the involvement of additional factors that impair growth (e.g., coenzyme A (CoA), see below). Disconnection between cell growth and peptidoglycan synthesis has been previously observed [44].

Altogether, these results strongly suggest that KG accumulation can specifically affect cell morphogenesis by reducing PG precursor synthesis.

### A reduction in CoA levels contributes to KG accumulation in the Δ*hfq* strain

What could cause the increased levels of KG when Hfq is absent? In various microorganisms, KG has been shown to accumulate under nitrogen starvation conditions [38, 48–50]. However, it is unlikely that the Δ*hfq* strain was deprived of nitrogen as cells were grown in PYE, a growth medium that contains amino acids as sources of nitrogen.

**Fig 6:**
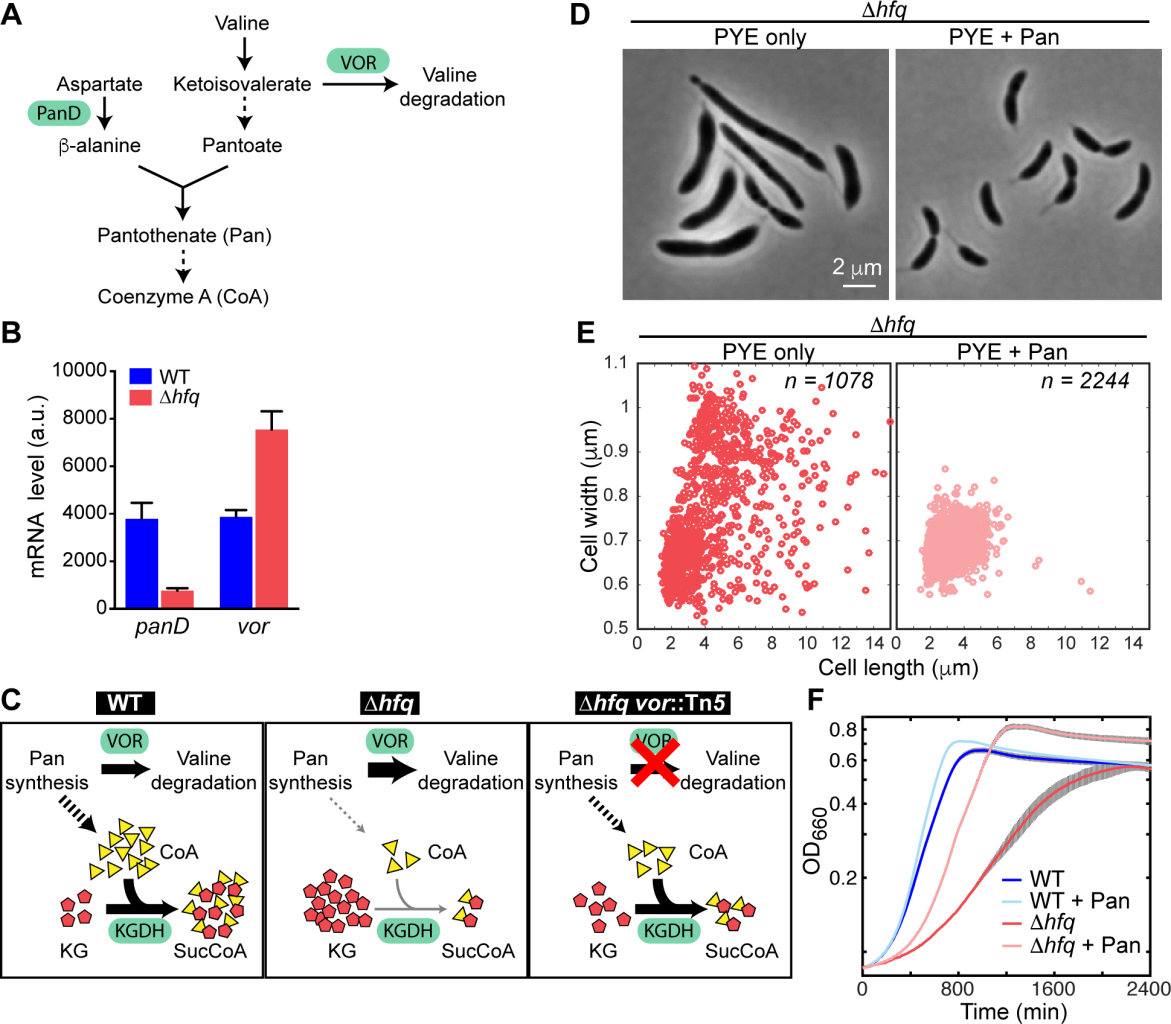
Link between the Δ*hfq* phenotypes and the biosynthesis of pantothenate and CoA. (A) Schematic of the pantothenate and CoA biosynthesis pathway in *C*. *crescentus*. The solid arrows represent a single enzymatic step, while the dashed arrows denote multiple enzymatic steps. PanD and VOR are the only two enzymes in this pathway whose mRNA levels were affected by the *hfq* deletion. (B) *panD* and *vor* mRNA levels in the WT and Δ*hfq* strains measured in RNA-Seq experiments. The error bars represent the standard deviations from 3 biological replicates. (C) Proposed mechanism underlying KG accumulation in Δ*hfq* cells. Downregulation of *panD* expression and upregulation of *vor* expression lead to reduction in CoA abundance. In turn, lower CoA level reduces KGDH activity leading to KG accumulation. Inactivation of *vor* in the Δ*hfq vor*::Tn*5* suppressor strain partially restores CoA synthesis and improves KGDH activity, resulting in lower KG levels. (D) Phase contrast images of Δ*hfq* cells grown in PYE with or without 1 mM pantothenate (Pan) for 20 h at 30°C. (E) Scatter plots of cell lengths and widths of populations described in (D). (F) Growth curves of WT and Δ*hfq* strains cultured at 30°C in PYE with and without 1 mM Pan. Each curve represents the average of 3 replicates with the standard deviation shown in grey.

To gain more insight into the physiological changes associated with the *hfq* deletion, we performed an RNA-Seq experiment to compare the gene expression profiles of WT and Δ*hfq* cells grown in PYE cultures. Transcriptomic analysis revealed that hundreds of genes are differentially expressed by a fold-change ≥ 2 (*p*-value ≤ 0.01) in the absence of Hfq (S4A Table). These genes were associated with a wide range of cellular functions, as shown by the analysis of ‘clusters of orthologous groups’ (COGs) (S11 Fig). We specifically looked for differentially expressed genes whose functions could be tied to KG metabolism. Based on this analysis, we hypothesized that the KG accumulation in Δ*hfq* cells primarily stems from misregulation of two metabolic genes, *panD* (*CCNA_02380*) and *vor*. Both genes are involved in CoA metabolism (Fig 6A), which is directly tied to KG metabolism (Fig 3B). *panD*, which encodes an enzyme involved in β-alanine synthesis, was one of the most downregulated genes in the Δ*hfq* transcriptome with ~4.5-fold decrease (Fig 6B, S4 Table). β-alanine is a precursor for pantothenate, which is required for the synthesis of CoA. Expression of *vor* was, in contrast, elevated 2-fold in the absence of Hfq (Fig 6B). One of the predicted substrates of VOR, ketoisovalerate, is also involved in pantothenate biosynthesis. Hence, overexpression of VOR may draw more ketoisovalerate into the valine degradation pathway, potentially reducing pantothenate synthesis.

The combination of higher *vor* and lower *panD* expression levels in the Δ*hfq* strain may result in reduction of free CoA inside cells (Fig 6C). This prediction is consistent with the ~2-fold reduction in CoA levels in the absence of Hfq (Fig 3A, S3A Table). Reduced levels of CoA in Δ*hfq* cells could negatively affect the activity of KGDH, which uses CoA as a cofactor to convert KG into SucCoA, resulting in KG accumulation (Fig 6C) and cellular defects. This would explain why inactivation of *vor*, which is commonly found among the Tn*5* suppressors (Fig 2C), partially rescues the Δ*hfq* phenotypes; *vor* inactivation would increase the flux of ketoisovalerate into pantothenate synthesis, thereby making CoA available for KGDH, leading to a decrease in KG accumulation in the suppressor strain (Fig 6C). Further evidence of CoA limitation in Δ*hfq* cells was provided by the observation of an accumulation of pyruvate (Fig 3A, S3A Table), a substrate of the pyruvate dehydrogenase complex, which also requires CoA. Pyruvate levels were conversely lower in the suppressor *hfq vor*::Tn*5* strain compared to Δ*hfq* (Fig 3A, S3A Table).

This model predicts that increasing CoA levels would restore KG levels and rescue the Δ*hfq* defects. One way to increase the intracellular concentration of CoA is by supplementing the culture medium with the CoA precursor pantothenate, which can be taken up by many bacteria [51, 52]. Accordingly, Δ*hfq* cells grown in PYE with pantothenate (1 mM) displayed a decrease in KG abundance (Fig 3C) and a restoration of the WT morphology (Fig 6D and 6E, S1 Table).

Interestingly, growth of the *hfq* mutant was also improved with pantothenate supplementation (Fig 6F). This observation suggests that both growth and morphology phenotypes of the Δ*hfq* strain originate from metabolic perturbations in general and the reduction in CoA levels in particular. The two phenotypes are, however, uncoupled downstream of the metabolic dysregulation, as shown by the addition of DAP, which exclusively suppresses the cell shape defects by restoring PG precursor synthesis (Fig 5E and 5F).

### Metabolic perturbation of PG synthesis leads to increased antibiotic susceptibility

PG is a common target for many successful antimicrobial agents [53]. Therefore, we reasoned that the metabolism-dependent reduction of PG precursor synthesis in the Δ*hfq* strain may also alter the susceptibility of the cell to PG-targeting antibiotics. To test this hypothesis, we used disk diffusion assays on PYE agar plates to quantify the sensitivity of Δ*hfq* and control strains to three PG-targeting antibiotics. Specifically, we used fosfomycin, cephalexin, and vancomycin (*C*. *crescentus* is sensitive to vancomycin despite having an outer membrane [54]), which target different stages of PG synthesis. As mentioned above, fosfomycin inhibits the synthesis of PG precursors in the cytoplasm (Fig 5A) [55]. In contrast, cephalexin and vancomycin inhibit later steps in PG assembly that occur in the periplasm [56, 57].

**Fig 7:**
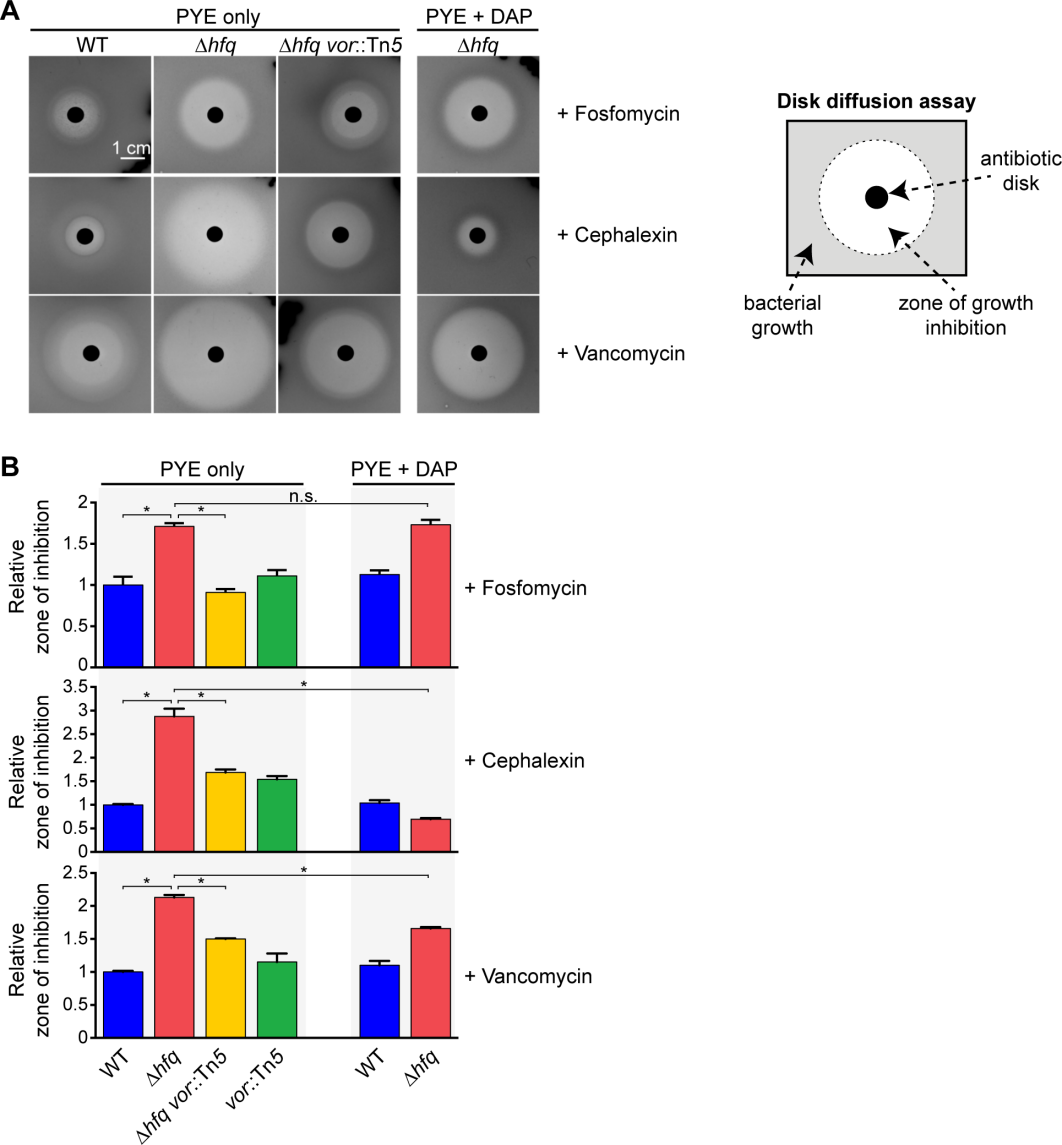
Deletion of *hfq* alters cell sensitivity to PG-targeting antibiotics. (A) Representative images from disk diffusion assays evaluating the sensitivity of Δ*hfq* and control strains toward fosfomycin, cephalexin, and vancomycin. Cells from exponentially growing cultures were mixed with PYE soft agar (0.75%) and poured on top of PYE agar plates in the absence or presence of 100 μM DAP. Plates were incubated at 30°C for 70 h with antibiotic-loaded filter disks. (B) Quantification of antibiotic sensitivity for Δ*hfq* and control strains, as determined by the diameter of the clear zone of inhibition around the disk relative to the zone of inhibition for the WT strain. The error bars represent the standard deviations from 3 independent experiments. “n.s.”, not significant (*p*-value > 0.05); *, *p*-value < 0.01 by two-tailed *t*-test.

We found that the Δ*hfq* strain exhibits increased sensitivity to all three PG-targeting antibiotics, as shown by the larger zones of inhibition relative to the wild-type strain (Fig 7A and 7B). Such increase in sensitivity was not observed for protein synthesis inhibitors, such as gentamicin and spectinomycin (S12 Fig). The hypersensitivity to PG-targeting antibiotics was partially suppressed in the Δ*hfq vor*::Tn*5* suppressor strain (Fig 7B), consistent with the partial rescue of PG precursor synthesis and the lower levels of KG in this strain (Figs 3C and 5D). In addition, supplementation of DAP in the plate reduced the heightened sensitivity of the Δ*hfq* strain to cephalexin and vancomycin, while exhibiting no effect on the susceptibility of the WT strain (Fig 6B). Interestingly, we did not observe any change in Δ*hfq* sensitivity to fosfomycin when grown in the presence of DAP (see Discussion).

Altogether, these results suggest that the reduction of PG precursor synthesis due to metabolic perturbations in the Δ*hfq* strain impairs cell wall biogenesis and increases the cell’s susceptibility to PG-targeting antibiotics.

### The Hfq phenotypes are sensitive to nutrient availability

So far, we have examined the *hfq* deletion phenotypes during growth on amino acids as the main carbon sources. Microbes are often faced with different nutrient sources that can have a significant influence on cellular metabolism. Therefore, we hypothesized that the nutrient composition of the growth medium would affect, positively or negatively, the Δ*hfq* phenotypes.

Aside from amino acids, glucose and xylose are the best characterized carbon sources for *C*. *crescentus* growth in the laboratory [36]. In glucose-containing medium (M2G), Δ*hfq* mutant cells grew slower than wild-type cells, but their morphology appeared normal (S13A–S13C Fig, S1 Table). Accordingly, metabolomic analysis of M2G-grown Δ*hfq* cells showed perturbations in the levels of central metabolites, but no significant accumulation of KG (S3B Table). Growth in xylose as a carbon source (M2X) aggravated the fitness defect associated with the *hfq* deletion, as Δ*hfq* cells could barely grow in M2X (S13D Fig) and appeared stressed as suggested by the prevalence of storage granules (S13E Fig, arrow). Investigating the effect of xylose metabolism on cell morphology was problematic, as growth is required to observe a dramatic cell shape defect. However, despite the small amount of growth, the few Δ*hfq* cells in the M2X culture already presented a cell widening phenotype relative to WT (S13E and S13F Fig, S1 Table). The detrimental effects of xylose on Δ*hfq* cells provide a possible explanation for the annotation of *hfq* as an essential gene in the genome-wide Tn-Seq study, as the growth medium used in this study contained xylose [26].

Altogether, the strong phenotypic sensitivity of the Δ*hfq* mutant to the available nutrient source further supports the notion that maintaining metabolic homeostasis is not only important for optimal cell growth but also for proper cell morphogenesis in different nutritional environments.

## Discussion

**Fig 8:**
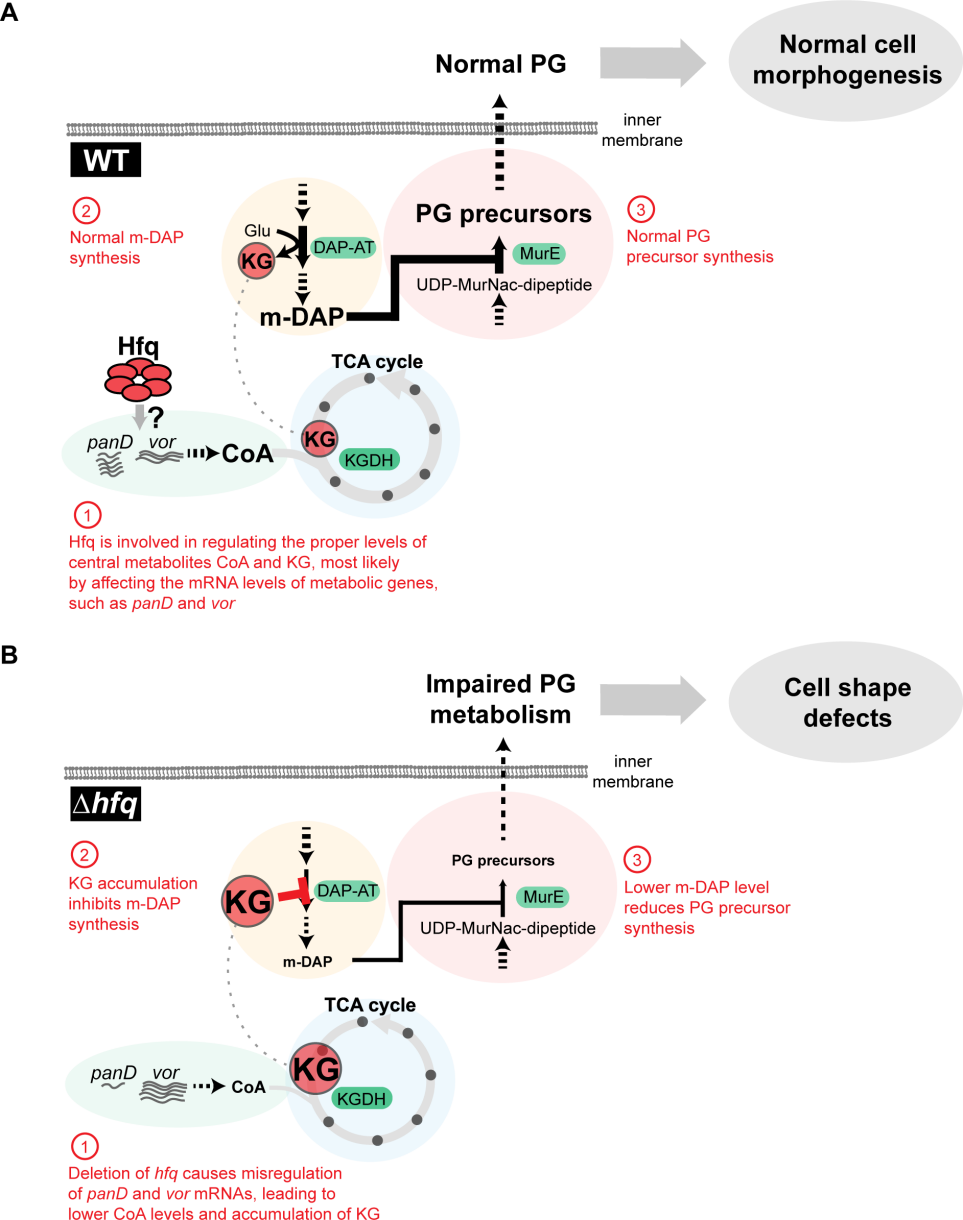
Proposed model for the crosstalk between central metabolism and PG precursor synthesis. (A) In wild-type *C*. *crescentus* grown in PYE medium, Hfq is involved in maintaining the homeostasis of central metabolites, such as CoA and KG, presumably by affecting the mRNA levels of metabolic genes *panD* and *vor*. KG homeostasis is important to prevent interference with the m-DAP and PG biosynthesis pathways and to maintain normal cell morphogenesis. The solid arrows represent a single enzymatic step, while the dashed arrows denote multiple enzymatic steps. (B) In the absence of Hfq, the mRNA levels of *panD* and *vor* are altered, leading to reduced CoA levels and accumulation of KG. High level of KG inhibits m-DAP synthesis, which, in turn, reduces the production of PG precursors and causes cell shape defects.

Our work demonstrates the importance of Hfq in regulating central metabolism in *C*. *crescentus* (Figs 2, 3, 6 and 8, S13 Fig). The key finding is the direct connection between the TCA cycle and PG synthesis, which provides an explanation for the cell shape defects in Δ*hfq* cells grown in PYE. Based on RNA-Seq data, Hfq does not regulate the expression of genes known to be involved in cell shape regulation (S4 Table). Instead, our data support a model for a metabolite-dependent regulation of PG synthesis (Fig 8). In this model, imbalance of a TCA cycle metabolite directly affects a PG biosynthetic step.

The precise mechanism by which Hfq affects the TCA cycle is unknown. As an RNA chaperone, Hfq regulates gene expression at the posttranscriptional level, typically in conjunction with small regulatory RNAs (sRNAs) [14–16]. We propose that the metabolic perturbations in Δ*hfq* cells are connected to the misregulated expression of metabolic genes (e.g., *panD*, *vor*) (Fig 8), perhaps through the loss of sRNA activity. While the mechanistic details for this regulation remain to be determined, the proper control of metabolic genes is critical for maintaining the correct balance of central metabolites. Disruption of KG homeostasis in the Δ*hfq* mutant is likely due to a block in the TCA cycle caused by a reduction in CoA level (Fig 8). The resulting accumulation of KG inhibits DAP-AT enzymatic activity, limiting m-DAP synthesis, and thereby reducing the level of PG precursors (Fig 8). KG has recently emerged as an important molecule in bacteria that affects many cellular processes, including fatty acid metabolism, nitrogen metabolism, and sugar uptake [58]. Our work expands the list of cellular processes influenced by KG to include PG synthesis.

It might seem counterintuitive that reduced PG precursor synthesis would cause cells to become wider and more elongated. Recent work suggests that the increase in cell size upon inhibition of PG synthesis by fosfomycin is due to an imbalance between the growth of cell surface area and volume [44]. In this model, volume growth (due to the synthesis of cytoplasmic components) is faster than surface growth (synthesis of PG), potentially causing higher turgor pressure that leads to a cell width increase. In addition, the slower PG synthesis is proposed to trigger a delay in cell division, which presumably allows cells to accumulate sufficient PG materials, thereby leading to a cell length increase. A similar model may be applicable to the Δ*hfq* phenotypes since PG synthesis is also impaired in this strain. The reason for the large variation in cell size and shape defects within a clonal population of Δ*hfq* cells (Fig 1C) is unknown. This cell size variability can be rescued by the addition of DAP (Fig 5E and 5F), suggesting a potential heterogeneity in PG synthesis among Δ*hfq* cells.

A link between the TCA cycle and PG synthesis was previously noted in studies investigating *E*. *coli* mutants that require lysine supplementation when grown on glucose as the sole carbon source [59–61]. The lysine auxotrophy was proposed to stem from depletion of SucCoA, a cofactor required for the synthesis of m-DAP and lysine (Fig 5A) [60, 62, 63]. We found that in Δ*hfq* cells, the abundance of SucCoA is modestly decreased compared to the WT situation (Fig 3A, S3A Table). Therefore, it is possible that the lower SucCoA level may also contribute to the defective PG synthesis in the *hfq* mutant.

Central metabolism provides energy and building blocks for the cell. Therefore, maintaining the proper concentration of central metabolites—i.e., achieving homeostasis—is important for driving enzymatic reactions and achieving optimal growth under various conditions. What is less appreciated is that central metabolites, of which KG is a prime example, can be involved in a wide range of enzymatic reactions inside cells. In addition to serving as substrates in their primary pathways, these metabolites can be by-products of reactions in other pathways. An excess of these common metabolites will have unanticipated inhibitory effects on such pathways, as is the case for KG on m-DAP synthesis (Figs 5A and 8). Our work illustrates the importance of homeostatic control of central metabolites in preventing a “ripple effect” through interference with other cellular processes.

The observation that KG accumulation causes not only cell shape defects, but also increased sensitivity to PG-specific antibiotics exemplifies a detrimental consequence of this ripple effect (Fig 7). DAP supplementation to Δ*hfq* cultures, which restores PG synthesis, reversed the hypersensitive phenotypes toward cephalexin and vancomycin. Surprisingly, it did not suppress the fosfomycin hypersensitivity. The basis for this discrepancy is unclear. Since fosfomycin treatment downregulates the expression of genes encoding various transporters for amino acids, sugars, and cations in *Staphylococcus aureus* [64], it is possible that fosfomycin may interfere with the uptake of DAP in *C*. *crescentus*. Restoring PG precursor synthesis through a genetic mutation, such as in the Δ*hfq vor*::Tn*5* strain, was sufficient to reduce fosfomycin sensitivity (Fig 7). Thus, the hypersensitivity to fosfomycin is linked to the impaired PG synthesis, similar to what we observed for cephalexin and vancomycin. Importantly, the fact that the antibiotic susceptibilities of Δ*hfq* cells are linked to TCA cycle perturbations further supports the growing notion that the state of cellular metabolism is an important determinant for antibiotic efficacy [65–67]. Since the TCA cycle and the PG synthesis pathway described here are broadly conserved in bacteria, our results suggest a potential new strategy for combination drug therapy that exploits an accumulation in KG to potentiate the action of antibiotics targeting PG-related processes.

## Materials and methods

### Strains, plasmids and oligonucleotides

The strains and plasmids used in this study are listed in S5 Table, and the details of their construction are described in S6 Table. The list of oligonucleotides used in this study is in S7 Table.

### Growth conditions and measurements

Unless otherwise indicated, *C*. *crescentus* strains were cultured at 30°C in PYE broth (2 g/L bacto peptone, 1 g/L yeast extract, 1 mM MgSO_4_, 0.5 mM CaCl_2_). For experiments performed in defined media, *C*. *crescentus* was grown in M2 medium (0.87 g/L Na_2_HPO_4_, 0.54 g/L KH_2_PO_4_, 0.5 g/L NH_4_Cl, 0.5 mM MgSO_4_, 0.5 mM CaCl_2_, 0.01 mM FeSO_4_) with either 0.2% (weight/volume, w/v) glucose (M2G), 0.2% (w/v) xylose (M2X), or a mixture of 2 mM leucine, isoleucine, and valine (M2BCAA). Unless indicated otherwise, M2BCAA is supplemented with 1x Kao and Michayluk vitamin mix (K3129, Sigma-Aldrich). When appropriate, vanillic acid (0.05 or 0.5 mM) was added as indicated. Antibiotics used for *C*. *crescentus* growth were as follows: kanamycin 5 μg/mL, oxytetracycline 1 μg/mL, spectinomycin 25 μg/mL, gentamicin 2 μg/mL. For growth curve measurements, overnight cultures were diluted to an OD_660_ ~0.01 in the appropriate growth medium. The diluted cultures (200 μL) were then transferred into 96-well plates and grown at 30°C in a Synergy2 microplate reader (BioTek). Growth curves were generated by reading OD_660_ every 10 min for 40–48 h. Growth rates were determined by fitting an exponential function to the early phase of the growth curves (up to OD_660_ = 0.2). When appropriate, vanillic acid and other metabolites were added at the beginning of the growth curve measurements.

### Microscopy and image post-processing analysis

Prior to imaging, *C*. *crescentus* cells were cultured to an OD_660_ ~0.3 (corresponding to exponential phase) at 30°C and spotted on 1% agarose pads containing the same growth medium. Microscopy was performed on an Eclipse Ti-E microscope (Nikon, Tokyo, Japan), equipped with Perfect Focus System (Nikon), a phase-contrast objective Plan Apochromat 100X/1.40 NA (Nikon), and an ORCA-Flash4.0 V2 Digital CMOS camera (Hamamatsu Photonics, Hamamatsu City, Japan). All images were acquired using MetaMorph software (Molecular Devices, Sunnyvale, CA, USA), and analyzed with Metamorph, Oufti [68] and MATLAB software (MathWorks).

### Whole genome sequencing

Genomic DNA (gDNA) was extracted using ChargeSwitch kit (Thermo Fisher Scientific) from overnight cultures of the Δ*hfq* strain (CJW5477) grown in PYE at 30°C following the manufacturer’s recommendation. Prior to library preparation, the quality of the gDNA was assessed by measuring A260/A280 and A260/A230 ratio with a NanoDrop device (Thermo Fisher Scientific) and by running the sample on Bioanalyzer (Agilent). Library preparation and sequencing were done by the Yale Center for Genome Analysis (YCGA) on a HiSeq2500 with 75 bp paired-end reads. Data analysis to identify potential mutations was performed using breseq [69] against the *C*. *crescentus* CB15N reference genome (NC_011916.1). Whole genome sequencing result is available in the Sequence Read Archive database with ID# SRP105792.

### Tn*5* suppressor screen

EZ-Tn*5* transposome (R6Kγori/Kan-2, Epicentre) was used to mutagenize Δ*hfq* (CJW5477) cells. To introduce the transposon, 0.2 μL of EZ-Tn5 was electroporated into 50 μL of competent CJW5477 cells. PYE medium (1 mL) was added to the cells and the culture was incubated at 30°C for 1.5 h before being plated onto PYE plates supplemented with kanamycin to select for clones carrying the transposon. The plates were incubated at 30°C for 4–5 days prior to visual screening for clones that formed larger colonies. These potential ‘suppressors’ were also tested for oxytetracycline resistance to verify the presence of Δ*hfq*::*tet* allele. To map Tn*5* insertion sites, gDNA was extracted from 0.5 mL of overnight cultures growing in PYE medium using Puregene kit (Qiagen) following the recommended protocol. Two to four micrograms of gDNA were digested with either NcoI or SacII restriction enzymes (both enzymes cut gDNA outside of the transposon; New England Biolabs), circularized using T4 DNA ligase (New England Biolabs), electroporated into competent *E*. *coli* S17-1 λ*pir* or EC100D *pir-116* (Epicentre) cells and plated on LB agar plates supplemented with kanamycin to select for clones carrying circularized EZ-Tn*5* transposon. Plasmids were then extracted from the kanamycin resistant clones and sequenced using primers specific to the transposon as recommended by the EZ-Tn*5* transposome protocol (Epicentre).

### Gene repression using CRISPRi

The full details of the CRISPRi system are described in S1 Text.

### Metabolite extraction

For metabolite extraction from filter cultures, *C*. *crescentus* strains were grown at 30°C in the appropriate growth medium until reaching OD_660_ ~0.2–0.4. Approximately 1.5x10^9^ cells were transferred onto 0.22 μm nitrocellulose or polyethersulfone (PES) membrane filter (Millipore) by vacuum filtration. The filters were then deposited on the surface of agar made with the same growth medium, and the cells were allowed to continue growing at 30°C for 2 doublings. Metabolism was then quenched by dropping the filters directly into precooled acetonitrile/methanol/ H_2_O (40:40:20, kept at around -40°C). Cells were washed off the membrane filters and the entire solution was then subjected to mechanical lysis using 0.1 mm Zirconia beads in a Precellys tissue homogenizer (3 cycles of 15” on, 1’ off) maintained at 4°C. Lysates were clarified by centrifugation at 13,000 x g for 5 min and then filtered across a 0.22 μm Costar microcentrifuge nylon filter (Corning). Filtered metabolite extracts were kept at -80°C prior to analysis on LC-MS. Bacterial biomass of individual samples was determined for normalization by measuring the protein content of metabolite extracts using Pierce BCA assay kit (Thermo Fisher Scientific). For DapE depletion by CRISPRi, metabolite extracts were prepared directly from liquid cultures grown at 30°C in PYE medium in the presence or absence of 0.05 mM vanillic acid. Approximately 3x10^9^ exponentially growing cells were rapidly collected onto a 0.45 μm PES membrane filter (Millipore) by vacuum filtration, and metabolism was quenched by plunging the membrane filter into precooled acetonitrile/methanol/ H_2_O (40:40:20, kept at around -40°C). Metabolite extraction was then performed as described for the filtered cultures.

### Liquid chromatography-mass spectrometry (LC-MS) analysis

For LC-MS analysis, metabolite extracts were mixed in a 1:1 ratio with acetonitrile and 0.2% formic acid. After a centrifugation step (13,000 x g for 5 min), the extracts were analyzed on an Agilent 1200 liquid chromatography (LC) system with a Cogent Diamond Hydride type C column (MicroSolv Technology, Leland, NC, USA) coupled to an Agilent Accurate Mass TOF 6220 [70]. The mobile phase consisted of the following: solvent A (0.2% [v/v] formic acid and 99.8% H_2_O) and solvent B (0.2% [v/v] formic acid and 99.8% acetonitrile). The gradient used was as follows: 0–2 min, 85% solvent B; 3–5 min, 80% solvent B; 6–7 min, 75% solvent B; 8–9 min, 70% solvent B; 10–11.1 min, 50% solvent B; 11.1–14 min, 20% solvent B; and 14.1–24 min, 5% solvent B; followed by a 10 min re-equilibration period at 85% solvent B and a flow rate of 0.4 mL/min. Dynamic mass axis calibration was achieved by continuous infusion of a reference mass solution (mixture of acetic acid D4 and hexakis phosphazine). Metabolite identities were searched using a mass tolerance of <0.005 Da in Profinder 8.0 (Agilent). Metabolite levels were quantified by integrating the area under the peak, followed with normalization for protein concentration in the extract.

### KG quantification using an enzymatic assay

For this experiment, metabolite extracts were prepared from liquid cultures. *C*. *crescentus* strains were grown at 30°C in the appropriate growth media until reaching OD_660_ ~0.2–0.4 and ~1.5x10^9^ of cells were quickly collected onto a 0.45 μm PES membrane filter (Millipore) by vacuum filtration. The membrane filter was plunged into 0.5 M formic acid solution (kept at 4°C) to quench metabolism. Cells were washed off the membrane filters and vortexed briefly. The solution was kept at 4°C for 1 h. Lysates were clarified by centrifugation, frozen at -80°C and lyophilized. Metabolites were resuspended in 10 mM Tris-HCl pH 7.6, and KG was quantified using the KG assay kit (Sigma-Aldrich) following the manufacturer’s recommendation. The protein content in the extract was measured for normalization using the Pierce BCA assay kit (Thermo Fisher Scientific).

### Library preparation, sequencing and data analysis for RNA-Seq experiments

*C*. *crescentus* wild-type CB15N and Δ*hfq* (CJW5477) cells were grown, in triplicate, at 30°C in PYE until the cultures reached an OD_660_ between 0.2 and 0.3. At this point, 20–40 mL of culture were harvested by centrifugation at 4°C for 5 min at ~7000 x *g*. Total RNA was extracted using Trizol (Thermo Fisher Scientific) according to the manufacturer’s protocol, except that centrifugation was performed at ~21000 x *g*. RNA pellets were resuspended in 100 μL of DEPC water and incubated for 5 min at 55°C. Size and integrity of the extracted RNA were assessed by electrophoresis on denaturing agarose gel. Removal of contaminating DNA was done by treating ~10 μg of total RNA with 10 units of DNase I (Sigma-Aldrich) at 37°C following the manufacturer’s protocol. The reactions were subjected to phenol:chloroform extraction and ethanol precipitation to purify total RNA. DNA-free total RNA was further evaluated by absorbance ratio 260/280 nm and 260/230 nm using a Nanodrop device (Thermo Fisher Scientific). Samples were considered good if the ratio 260/280 nm was >1.9. rRNA depletion was performed using Ribo-zero rRNA removal kit for Gram-Negative bacteria (Illumina), as recommended by the manufacturer. RNA-Seq library was prepared using ScriptSeq v2 kit (Illumina) with multiplexing following the manufacturer’s recommendation. Sequencing was done at the Yale Center for Genome Analysis (YCGA) using HiSeq2000, 1x75 bp to generate ~30–50 million reads per sample. For data analysis, sequencing reads were trimmed using Cutadapt [71] and mapped onto the *C*. *crescentus* CB15N reference genome (NC_011916.1) using Bowtie2 [72]. The number of reads mapped to each gene was determined using HTSeq [73] and differential expression analysis was performed using DESeq2 [74]. The expression level for each gene was calculated as the number of reads mapped per kilobase of gene (‘gene count’) normalized by the 75^th^-percentile of all gene counts in the sample [75]. The raw RNA-Seq data is available from Gene Expression Omnibus (GEO) database with accession number GSE98467.

### Disk diffusion assay

Cells were grown in PYE medium to exponential phase (OD_660_ ~0.2) and 250 μL of culture was mixed with 4 mL of PYE soft agar (0.75% agarose, kept at 55–60°C) prior to being poured onto PYE agar plates (1.5% agarose) with or without 100 μM 2,6-l,l-diaminopimelate (cat # 89469, Sigma-Aldrich) supplementation. The plates were dried in the fume hood for at least 10 min before being used. Antibiotics were added onto sterile 6 mm filter disks (Sigma-Aldrich), dried in the fume hood for 10 min, and deposited on top of the soft agar plates. The plates were then incubated for 70 h at 30°C before measuring the zone of inhibition using a digital caliper. The reported numbers are the diameters of the clearing zone around the antibiotic-loaded filter disks. The total amount of antibiotic used per filter disk was as follows: fosfomycin 50 μg, cephalexin 50 μg, vancomycin 1 mg, gentamicin 100 μg, spectinomycin 100 μg.

### Quantitative real-time RT-PCR

Total RNA was extracted from 5–10 mL of cultures using Trizol (Thermo Fisher Scientific) according to the manufacturer’s protocol. Contaminating genomic DNA was removed by treating ~10 μg of total RNA with 2 units of TURBO DNase (Thermo Fisher Scientific), as recommended by the manufacturer. Quantitative real-time RT-PCR was performed with ~80 ng of total RNA using SYBR FAST One-Step qRT-PCR kit (Kapa Biosystems) following the manufacturer’s protocol. The cycling parameters used for these experiments were: 42°C for 5 min, 95°C for 3 min, 40 cycles of 95°C for 3 s and 60°C for 20 s, using BioRad CFX96 Real-Time PCR instrument. The level of *ftsZ* mRNA from each sample was normalized to the level of *pdhA* (*CCNA_01799*) mRNA (encoding the pyruvate dehydrogenase E1 subunit). Fold-change was calculated using the ΔΔCt method [76]. DNA oligonucleotides used for these experiments were irv1814/irv1815 for *ftsZ* and irv1771/irv1772 for *pdhA* (see S7 Table for the oligonucleotide sequences).

## Supporting information

S1 Fig Growth and morphology defects in the Δ*hfq* strain are not due to a polar effect on the expression of *hflX*. (A) Schematic of the deletion constructs for *hfq* and *hflX*. Each gene was separately replaced by an antibiotic resistance cassette (tetracycline resistance cassette for *hfq* and spectinomycin resistance cassette for *hflX*). (B) Growth curves of WT and Δ*hflX* strains grown in PYE medium at 30°C. Each curve represents the average of 3 replicates with the standard deviation shown in grey. (C) Phase contrast images of WT and Δ*hflX* cells from PYE cultures at 30ºC. (D) Scatter plots of cell lengths and widths of cell populations from (C).
(PDF)

S2 Fig The *vor*::Tn*5* and Δ*vor* alleles display similar phenotypes. (A) Schematics of the *vor*::Tn*5* and Δ*vor* constructs. The *vor*::Tn*5* construct contains a Tn*5* insertion at nucleotide position 875 in the *vor* coding region (corresponds to strain #6 in S2 Table). In the Δ*vor* construct, the entire coding region of *vor* is replaced with a spectinomycin resistance cassette. (B) Growth curves of WT, Δ*hfq*, and strains with various *vor* constructs grown in PYE medium at 30°C. Each curve represents the average of 3 replicates with the standard deviation shown in grey. (C and E) Phase contrast images of cells with *vor*::Tn*5* or Δ*vor* alleles from PYE cultures at 30°C. (D and F) Scatter plots of cell lengths and widths of cell populations from (C) and (E), respectively.
(PDF)

S3 Fig VOR is necessary for branched-chain amino acid (BCAA) utilization in *C. crescentus*. (A) Proposed BCAAs degradation pathway in *C*. *crescentus* based on BioCyc pathway annotation [34]. The genes encoding enzymes required in the pathway are shown. The reaction predicted to be catalyzed by VOR is shown in red. (B) Growth curves of WT and *vor*::Tn*5* strains in defined minimal medium with a mixture of leucine, isoleucine, and valine (2 mM each, M2BCAA) as carbon sources. (C) Growth curves of WT and *vor*::Tn*5* strains in defined minimal medium with glucose (0.2%, M2G). (D) Growth of WT and *vor*::Tn*5* strains in defined minimal medium (M2) in the presence or absence of BCAA and vitamin mix. Final OD_660_ (growth at saturation) was determined from cultures grown at 30ºC for 55 h in a 96-well plate. Error bars denote the standard deviation from 3 replicates. The dotted line denotes OD_660_ at the start of the measurements.
(PDF)

S4 Fig The enzymatic activity of VOR is required for the Δ*hfq* phenotypes. (A) Growth rates of the Δ*hfq* Δ*vor* double knockout strains carrying an empty plasmid (none), a plasmid encoding wild-type VOR (WT), or a plasmid encoding catalytically inactive VOR (E84A). The glutamate residue at position 84 of VOR is conserved in all TPP-utilizing enzymes [77–79]. A glutamate-to-alanine substitution at this position (E84A) has been shown to abolish enzymatic activity [80, 81] without affecting the overall structure of the protein [81]. These strains were grown in PYE at 30ºC with or without vanillic acid (50 μM), the inducer of VOR expression. Growth rates were calculated by fitting an exponential function to the growth curves. Error bars denote the standard deviation from 3 replicates. (B) Phase contrast images of Δ*hfq* Δ*vor* double knockout cells carrying plasmids encoding various VOR constructs grown in PYE at 30ºC for 20 h in the presence or absence of 50 μM vanillic acid. (C) Scatter plot of cell lengths and widths of cell populations described in (B).
(PDF)

S5 Fig Growth method for metabolomics. (A) Schematic of the metabolomics experiment. Exponentially growing cultures were deposited onto filter membranes and grown on top of solid PYE agar for 4 h (WT and *vor*::Tn*5*) or 7.5 h (Δ*hfq* and Δ*hfq vor*::Tn*5*), which corresponded to ~2 doubling times for each strain. Cells were quickly immersed into a mixture of acetonitrile/methanol/H_2_O (40:40:20) to rapidly stop metabolism, and were subjected to mechanical lysis. The lysates were clarified by centrifugation and filtration before LC-MS analysis. (B) Phase contrast images of WT and Δ*hfq* cells grown on filters deposited on top of PYE agar at 30°C for 4 h and 7.5 h, respectively. Cells were washed off the membrane filters and then imaged on 1% PYE agarose pads.
(PDF)

S6 Fig FtsZ depletion in *C. crescentus* using CRISPRi. (A) Time-course images for FtsZ depletion using CRISPRi. Cells were grown in PYE medium at 30°C until early exponential phase after which vanillic acid (0.5 mM) was added to induce dCas9 expression for depletion. The sgRNA targeting *ftsZ* was constitutively expressed. (B) Quantification of cell length distributions over time in cultures with (FtsZ depletion) or without (no depletion) vanillic acid. (C) Quantification of *ftsZ* mRNA levels by quantitative real-time RT-PCR following CRISPRi depletion. Cells were grown as described in (A). The levels of *ftsZ* mRNA are relative to mRNA levels before depletion (0 h). Error bars denote the standard deviation from 3 biological replicates.
(PDF)

S7 Fig Cell morphology phenotypes of *sucA* temperature sensitive (ts) strains. Phase contrast images of two independent strains harboring separate *sucA* ts alleles grown at permissive (28ºC) and restrictive (38ºC for 6 h) temperatures in PYE medium. Scatter plots of cell lengths and widths for each cell population are shown.
(PDF)

S8 Fig Cell morphology defects upon treatment with fosfomycin. Phase contrast images from WT cells grown in PYE at 30°C for 5 h in the presence or absence of 5 μg/mL fosfomycin. Scatter plots of cell lengths and widths for each cell population is shown.
(PDF)

S9 Fig Depletion of DapE causes an accumulation of UDP-MurNAc-dipeptide and changes in cell morphology. (A) LC-MS chromatogram showing the accumulation of UDP-MurNAc-dipeptide (m/z = 878.1751 ± 10 ppm, ~12.22 min) upon DapE depletion by CRISPRi. DapE was depleted by growing the CRISPRi strain (CJW5893) in PYE liquid cultures at 30ºC supplemented with 0.05 mM vanillic acid for 20 h. Metabolites were extracted directly from liquid cultures and subjected to LC-MS analysis. For comparison, metabolite extracts from WT and Δ*hfq* cells grown on membrane filters (as described for Fig 3) were analyzed on the same run. Traces from 3 biological replicates are shown for each strain and condition. (B) Phase contrast images of the CRISPRi strain with or without DapE depletion. The expression of dCas9 was induced with 0.05 mM vanillic acid, and cells were grown for 20 h in PYE at 30ºC before imaging. Scatter plots of cell length and width are shown.
(PDF)

S10 Fig Growth of WT and Δ*hfq* strains in the presence or absence of DAP. Growth curves of WT and Δ*hfq* cells at 30°C in 96-well plates with PYE medium with and without 100 μM DAP. Each curve represents the average of 3 replicates with the standard deviation shown in grey.
(PDF)

S11 Fig Deletion of *hfq* affects the expression of genes involved in various functions. Functional classification of the differentially expressed genes between WT and Δ*hfq* strains based on COG analysis. RNA-Seq experiment was performed on cells grown in liquid PYE cultures at 30°C. About 15% of the *C*. *crescentus* genome (572 genes out of 4086) was identified as differentially expressed (fold-change ≥ 2 and *p*-value ≤ 0.01) between the two strains using DESeq2 from 3 biological replicates.
(PDF)

S12 Fig Deletion of *hfq* does not substantially increase cell sensitivity to common translation inhibitors. Quantification of antibiotic sensitivity for Δ*hfq* and control strains toward gentamicin and spectinomycin. Cells from exponentially growing cultures were mixed with PYE soft agar (0.75%) and poured on top of PYE agar plates in the absence or presence of 100 μM DAP. Plates were incubated at 30°C for 70 h with antibiotic-loaded filter disks. Antibiotic sensitivity was measured as the diameter of the zone of growth inhibition around the disk relative to the zone of inhibition for the WT strain. The mean and standard deviation from 3 independent experiments are shown.
(PDF)

S13 Fig Growth and morphology phenotypes of Δ*hfq* cells grown with different carbon sources. (A) Growth curves of WT and Δ*hfq* cells at 30°C in a 96-well plate containing minimal medium with glucose as a sole carbon source (M2G). For the growth curves, each curve represents the average of 3 replicates with the standard deviation shown in grey. (B) Phase contrast images of WT and Δ*hfq* cells grown in M2G at 30°C. (C) The measurements of cell dimensions from populations in (B). (D-F) Similar to (A-C) with cells grown in minimal medium containing xylose (M2X). Since the growth of Δ*hfq* is severely inhibited in the presence of xylose, cells were grown in M2G before being diluted into M2X for growth measurements or imaging. Arrows denote the presence of granules in M2X-grown Δ*hfq* cells.
(PDF)

S1 Table Cell length and width measurements for various strains. (XLSX)

S2 Table Location of the Tn*5* insertion for the top 30 fastest growing Δ*hfq* suppressors. (XLSX)

S3 Table Metabolomics analysis of various *C. crescentus* strains. Metabolite abundance from WT, Δ*hfq*, Δ*hfq vor*::Tn*5*, and *vor*::Tn*5* cells grown in PYE (A) and M2G (B).
(XLSX)

S4 Table Transcriptomics analysis of WT and Δ*hfq* strains grown in PYE. (A) List of genes significantly affected by an *hfq* deletion in PYE. (B) Expression level of all genes in WT and Δ*hfq* strains grown in PYE.
(XLSX)

S5 Table Strains and plasmids used in this study. (DOCX)

S6 Table Construction of strains and plasmids used in this study. (DOCX)

S7 Table Oligonucleotides used in this study. (DOCX)

S1 Text CRISPRi for *C. crescentus*. (DOCX)
