# Supplementary figures and images for: Crosstalk between the tricarboxylic acid cycle and peptidoglycan synthesis in Caulobacter crescentus through the homeostatic control of α-ketoglutarate
Source: PLoS Genet. 2017 Aug 21;13(8):e1006978. doi: 10.1371/journal.pgen.1006978 (PMC5578688; doi:10.1371/journal.pgen.1006978)

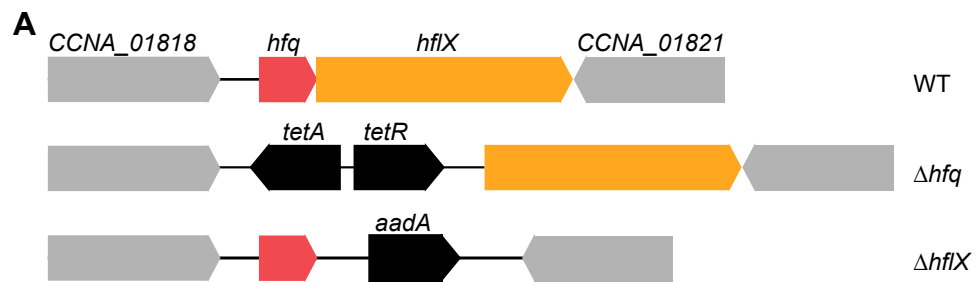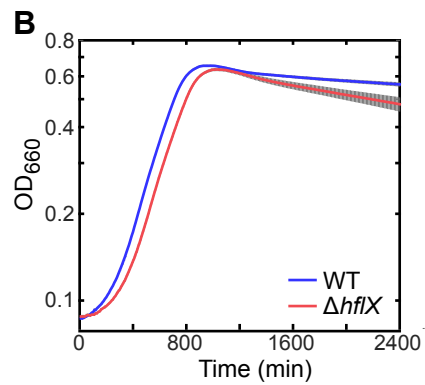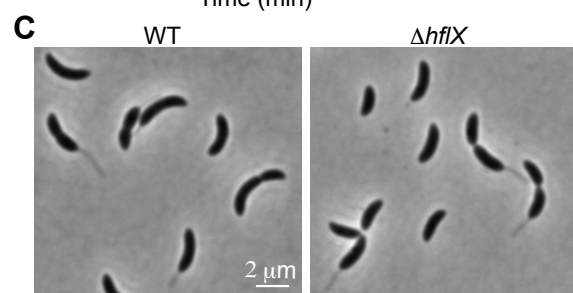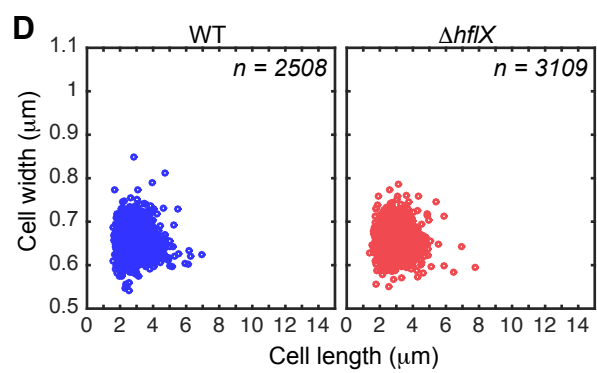

Supplement: S1 Fig — (A) Schematic of the deletion constructs for hfq and hflX. Each gene was separately replaced by an antibiotic resistance cassette (tetracycline resistance cassette for hfq and spectinomycin resistance cassette for hflX). (B) Growth curves of WT and ΔhflX strains grown in PYE medium at 30°C. Each curve represents the average of 3 replicates with the standard deviation shown in grey. (C) Phase contrast images of WT and ΔhflX cells from PYE cultures at 30ºC. (D) Scatter plots of cell lengths and widths of cell populations from (C). (PDF) [file pgen.1006978.s001.pdf]

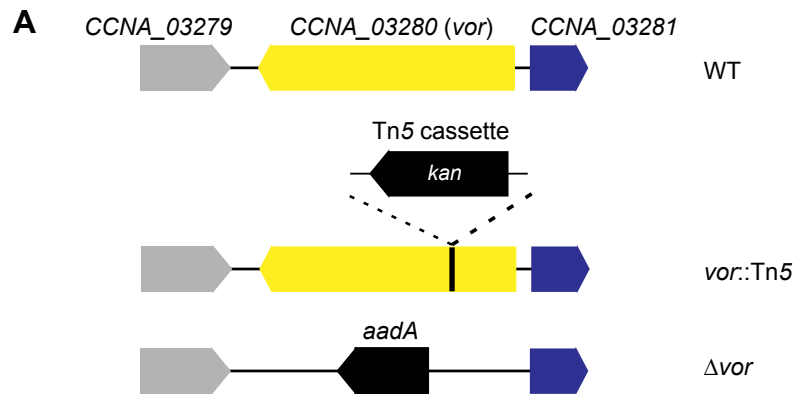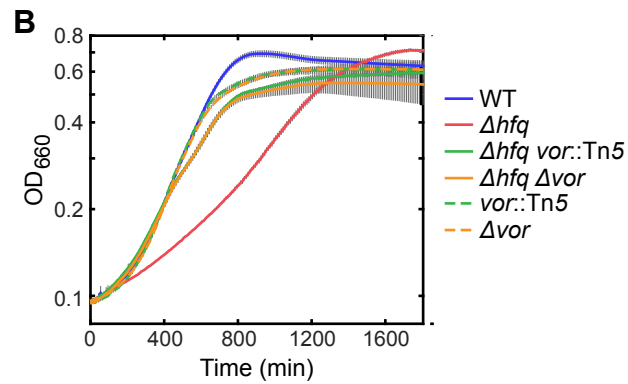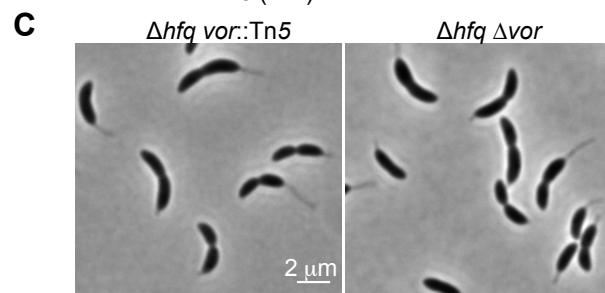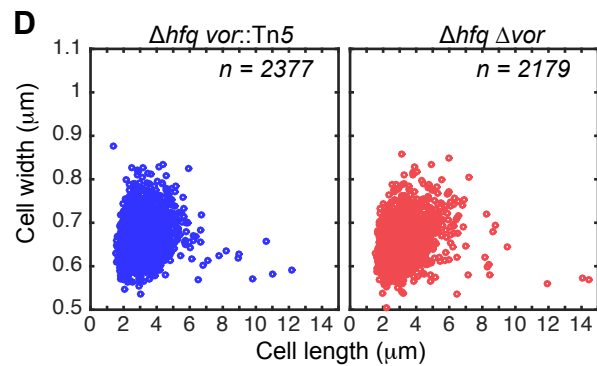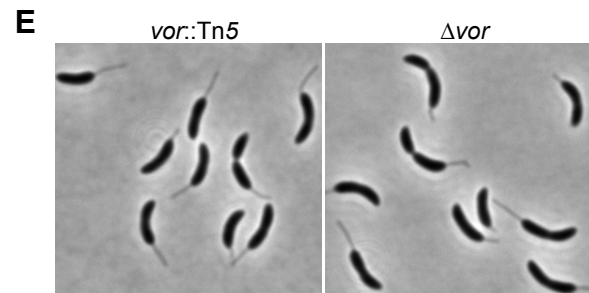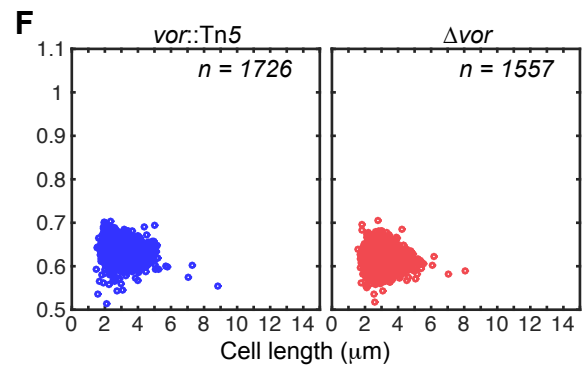

Supplement: S2 Fig — (A) Schematics of the vor::Tn5 and Δvor constructs. The vor::Tn5 construct contains a Tn5 insertion at nucleotide position 875 in the vor coding region (corresponds to strain #6 in S2 Table). In the Δvor construct, the entire coding region of vor is replaced with a spectinomycin resistance cassette. (B) Growth curves of WT, Δhfq, and strains with various vor constructs grown in PYE medium at 30°C. Each curve represents the average of 3 replicates with the standard deviation shown in grey. (C and E) Phase contrast images of cells with vor::Tn5 or Δvor alleles from PYE cultures at 30°C. (D and F) Scatter plots of cell lengths and widths of cell populations from (C) and (E), respectively. (PDF) [file pgen.1006978.s002.pdf]

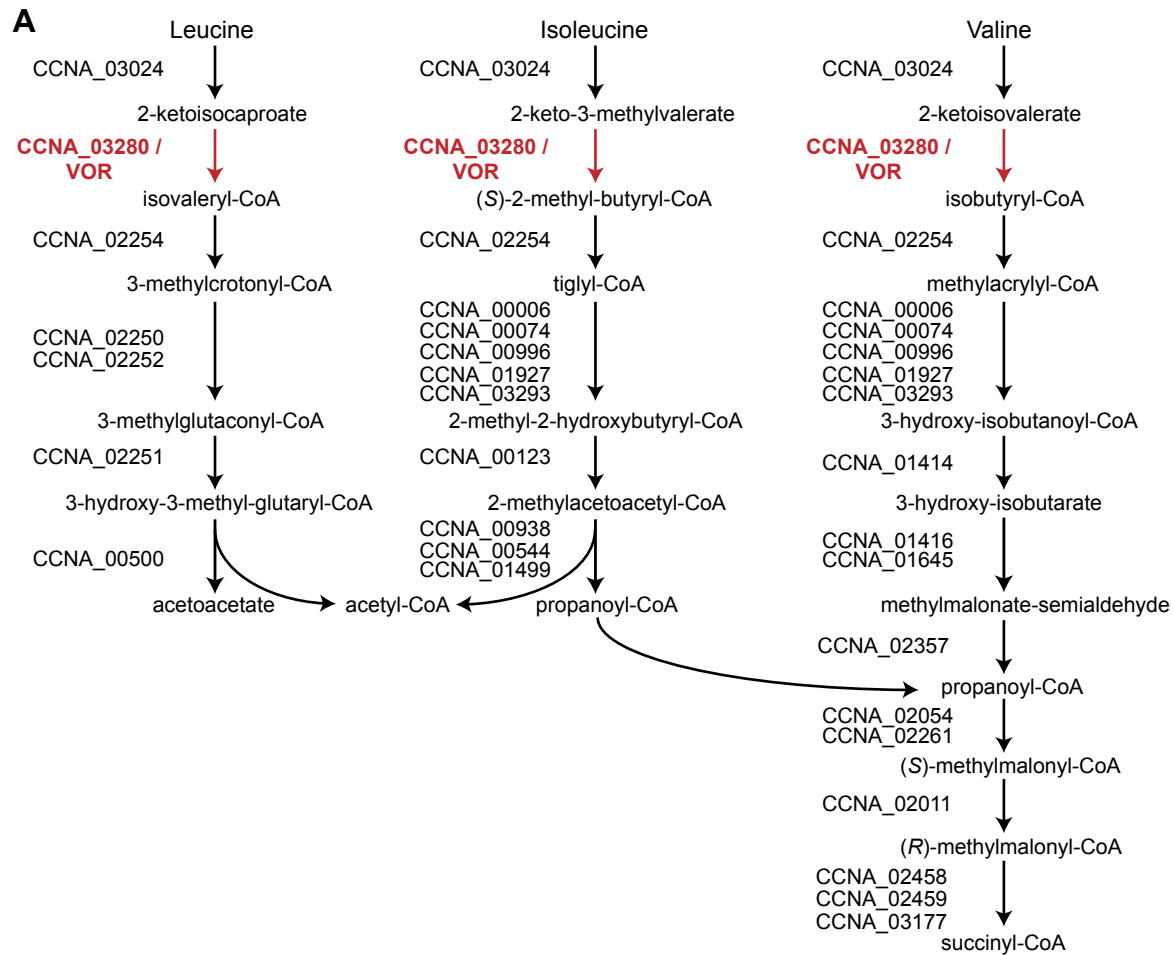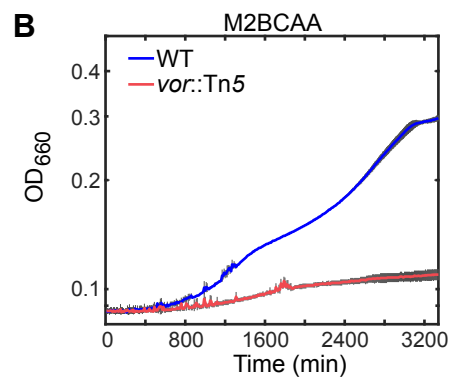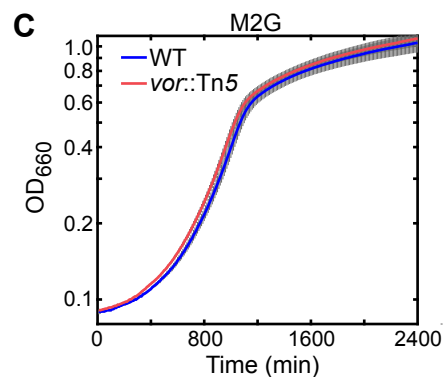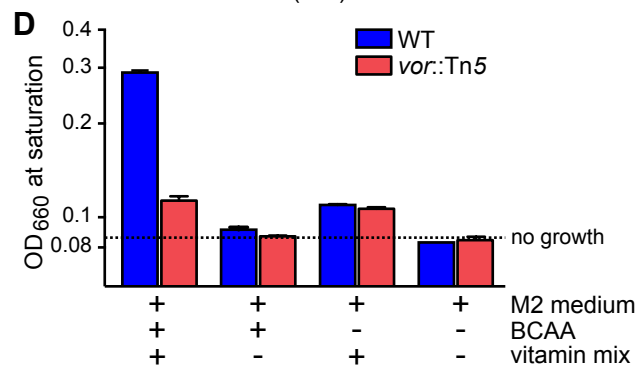

Supplement: S3 Fig — (A) Proposed BCAAs degradation pathway in C. crescentus based on BioCyc pathway annotation [34]. The genes encoding enzymes required in the pathway are shown. The reaction predicted to be catalyzed by VOR is shown in red. (B) Growth curves of WT and vor::Tn5 strains in defined minimal medium with a mixture of leucine, isoleucine, and valine (2 mM each, M2BCAA) as carbon sources. (C) Growth curves of WT and vor::Tn5 strains in defined minimal medium with glucose (0.2%, M2G). (D) Growth of WT and vor::Tn5 strains in defined minimal medium (M2) in the presence or absence of BCAA and vitamin mix. Final OD660 (growth at saturation) was determined from cultures grown at 30ºC for 55 h in a 96-well plate. Error bars denote the standard deviation from 3 replicates. The dotted line denotes OD660 at the start of the measurements. (PDF) [file pgen.1006978.s003.pdf]

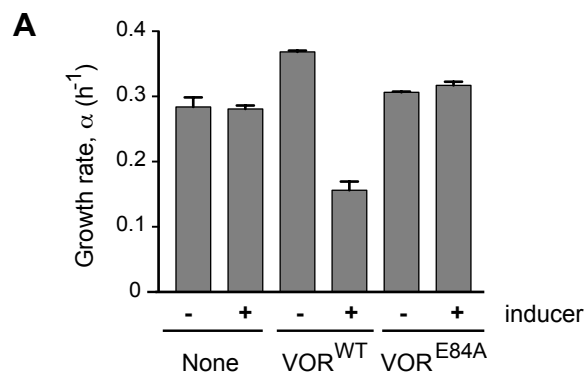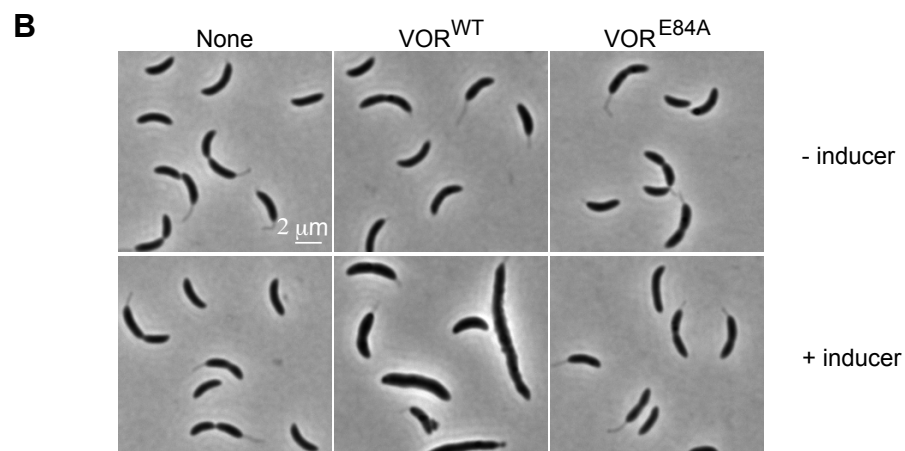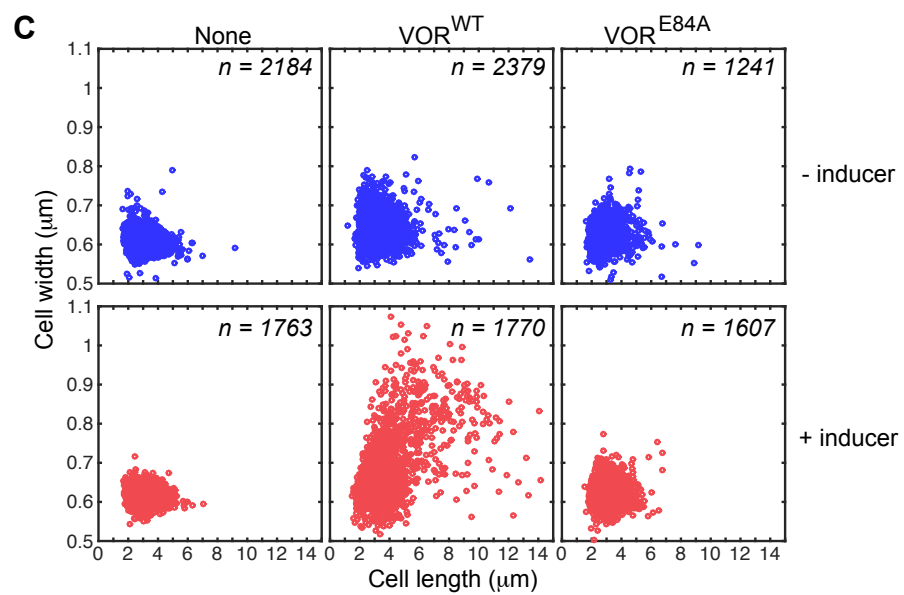

Supplement: S4 Fig — (A) Growth rates of the Δhfq Δvor double knockout strains carrying an empty plasmid (none), a plasmid encoding wild-type VOR (WT), or a plasmid encoding catalytically inactive VOR (E84A). The glutamate residue at position 84 of VOR is conserved in all TPP-utilizing enzymes [77–79]. A glutamate-to-alanine substitution at this position (E84A) has been shown to abolish enzymatic activity [80, 81] without affecting the overall structure of the protein [81]. These strains were grown in PYE at 30ºC with or without vanillic acid (50 μM), the inducer of VOR expression. Growth rates were calculated by fitting an exponential function to the growth curves. Error bars denote the standard deviation from 3 replicates. (B) Phase contrast images of Δhfq Δvor double knockout cells carrying plasmids encoding various VOR constructs grown in PYE at 30ºC for 20 h in the presence or absence of 50 μM vanillic acid. (C) Scatter plot of cell lengths and widths of cell populations described in (B). (PDF) [file pgen.1006978.s004.pdf]

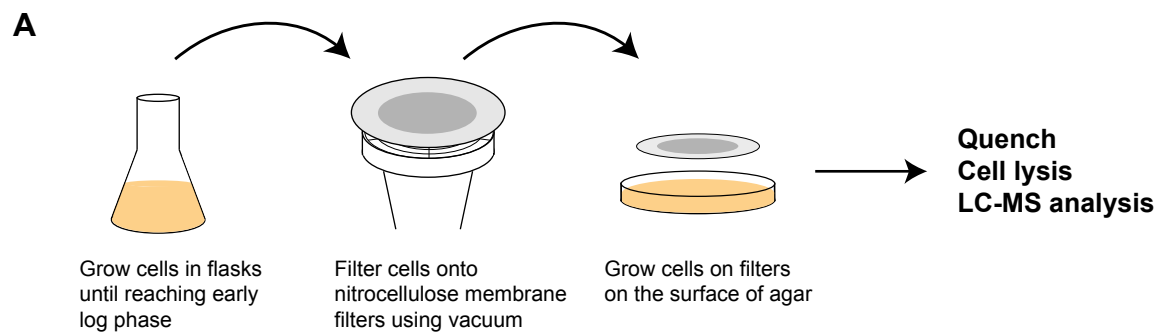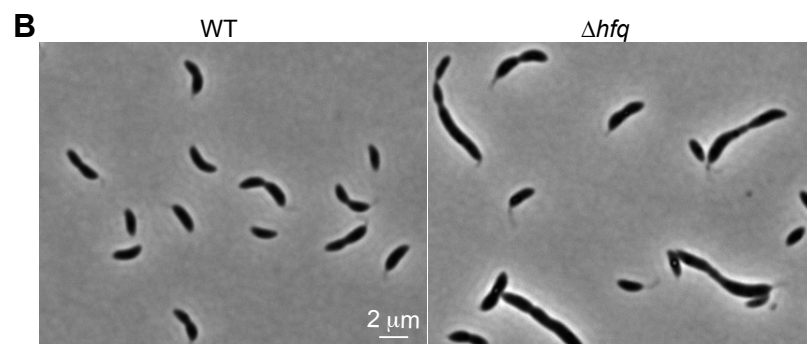

Supplement: S5 Fig — (A) Schematic of the metabolomics experiment. Exponentially growing cultures were deposited onto filter membranes and grown on top of solid PYE agar for 4 h (WT and vor::Tn5) or 7.5 h (Δhfq and Δhfq vor::Tn5), which corresponded to ~2 doubling times for each strain. Cells were quickly immersed into a mixture of acetonitrile/methanol/H2O (40:40:20) to rapidly stop metabolism, and were subjected to mechanical lysis. The lysates were clarified by centrifugation and filtration before LC-MS analysis. (B) Phase contrast images of WT and Δhfq cells grown on filters deposited on top of PYE agar at 30°C for 4 h and 7.5 h, respectively. Cells were washed off the membrane filters and then imaged on 1% PYE agarose pads. (PDF) [file pgen.1006978.s005.pdf]

**A** CRISPRi with sgRNA-ftsZ

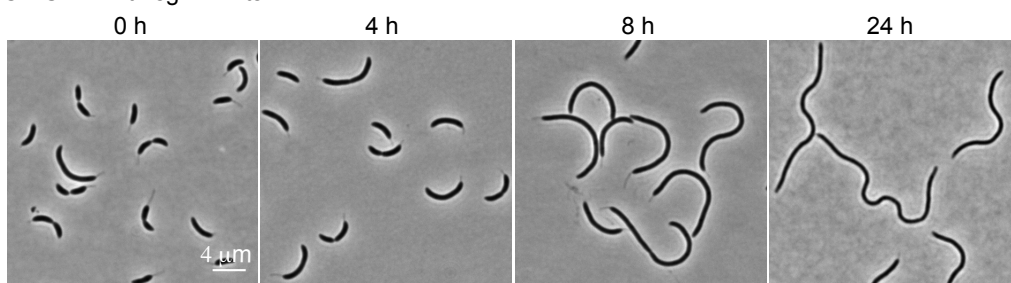

**B**

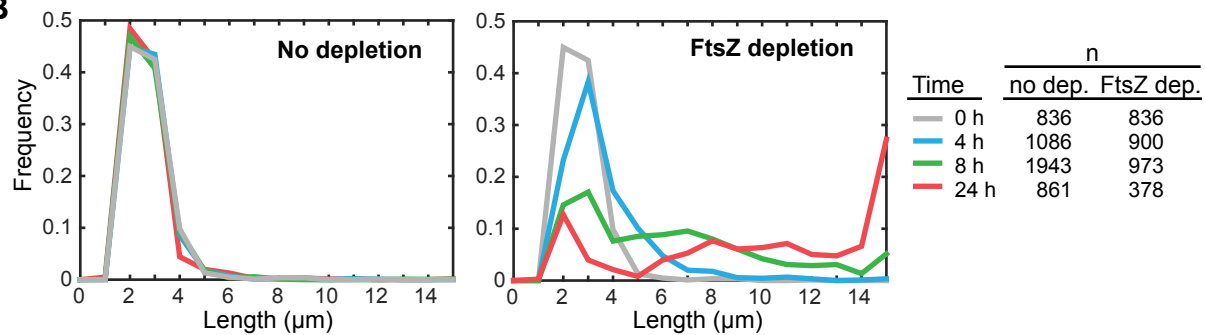

**C**

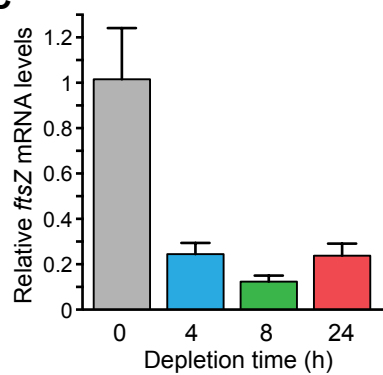

Supplement: S6 Fig — (A) Time-course images for FtsZ depletion using CRISPRi. Cells were grown in PYE medium at 30°C until early exponential phase after which vanillic acid (0.5 mM) was added to induce dCas9 expression for depletion. The sgRNA targeting ftsZ was constitutively expressed. (B) Quantification of cell length distributions over time in cultures with (FtsZ depletion) or without (no depletion) vanillic acid. (C) Quantification of ftsZ mRNA levels by quantitative real-time RT-PCR following CRISPRi depletion. Cells were grown as described in (A). The levels of ftsZ mRNA are relative to mRNA levels before depletion (0 h). Error bars denote the standard deviation from 3 biological replicates. (PDF) [file pgen.1006978.s006.pdf]

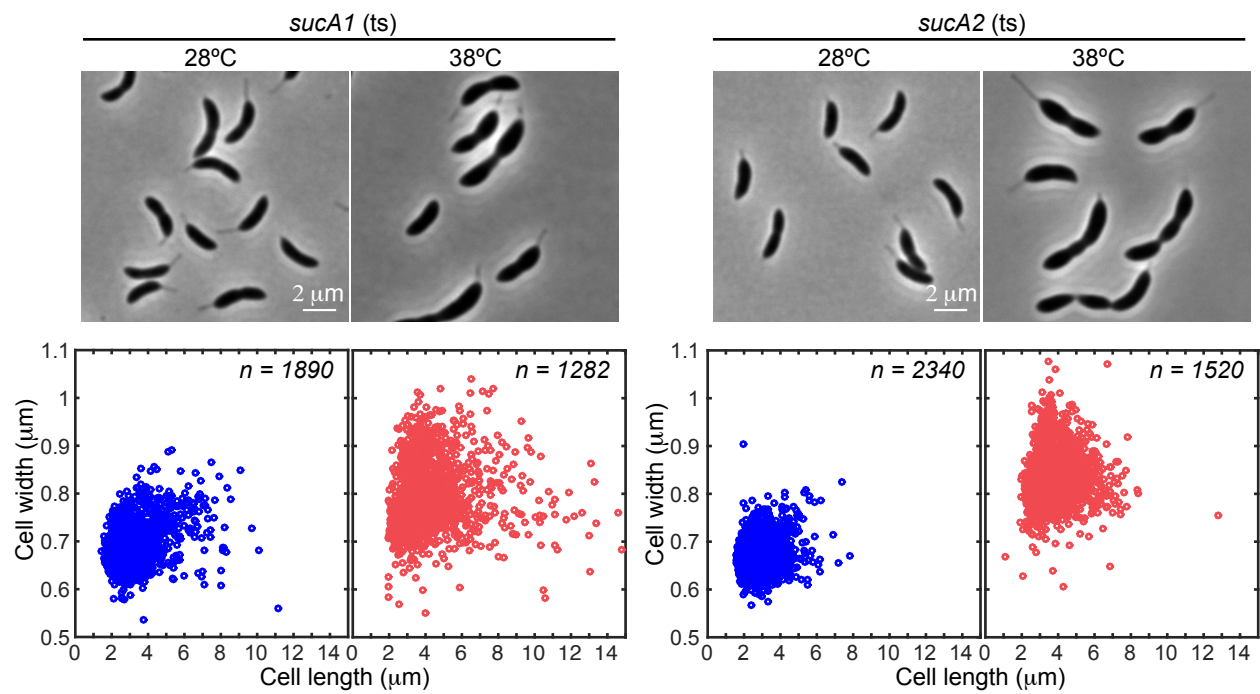

Supplement: S7 Fig — Phase contrast images of two independent strains harboring separate sucA ts alleles grown at permissive (28ºC) and restrictive (38ºC for 6 h) temperatures in PYE medium. Scatter plots of cell lengths and widths for each cell population are shown. (PDF) [file pgen.1006978.s007.pdf]

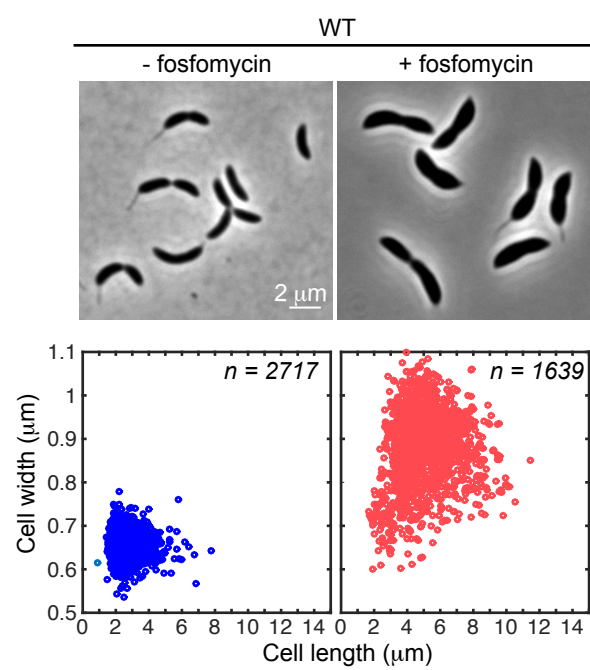

Supplement: S8 Fig — Phase contrast images from WT cells grown in PYE at 30°C for 5 h in the presence or absence of 5 μg/mL fosfomycin. Scatter plots of cell lengths and widths for each cell population is shown. (PDF) [file pgen.1006978.s008.pdf]

**A**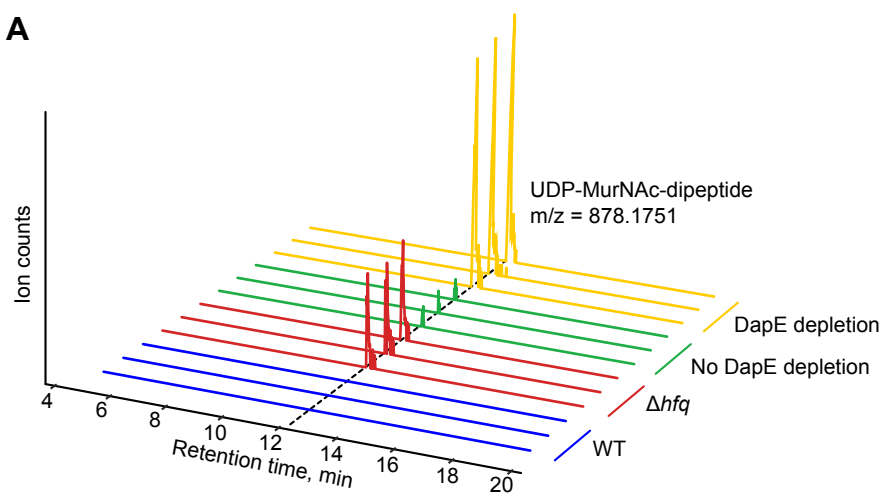**B**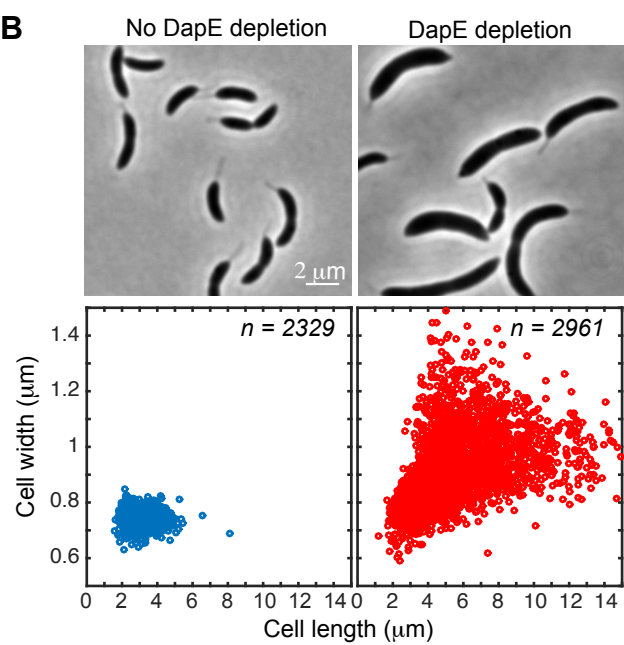

Supplement: S9 Fig — (A) LC-MS chromatogram showing the accumulation of UDP-MurNAc-dipeptide (m/z = 878.1751 ± 10 ppm, ~12.22 min) upon DapE depletion by CRISPRi. DapE was depleted by growing the CRISPRi strain (CJW5893) in PYE liquid cultures at 30ºC supplemented with 0.05 mM vanillic acid for 20 h. Metabolites were extracted directly from liquid cultures and subjected to LC-MS analysis. For comparison, metabolite extracts from WT and Δhfq cells grown on membrane filters (as described for Fig 3) were analyzed on the same run. Traces from 3 biological replicates are shown for each strain and condition. (B) Phase contrast images of the CRISPRi strain with or without DapE depletion. The expression of dCas9 was induced with 0.05 mM vanillic acid, and cells were grown for 20 h in PYE at 30ºC before imaging. Scatter plots of cell length and width are shown. (PDF) [file pgen.1006978.s009.pdf]

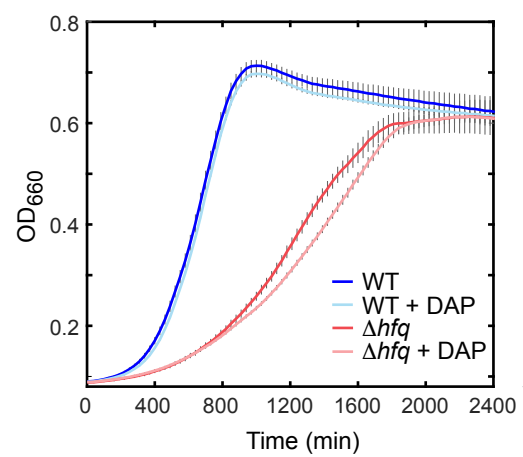

Supplement: S10 Fig — Growth curves of WT and Δhfq cells at 30°C in 96-well plates with PYE medium with and without 100 μM DAP. Each curve represents the average of 3 replicates with the standard deviation shown in grey. (PDF) [file pgen.1006978.s010.pdf]

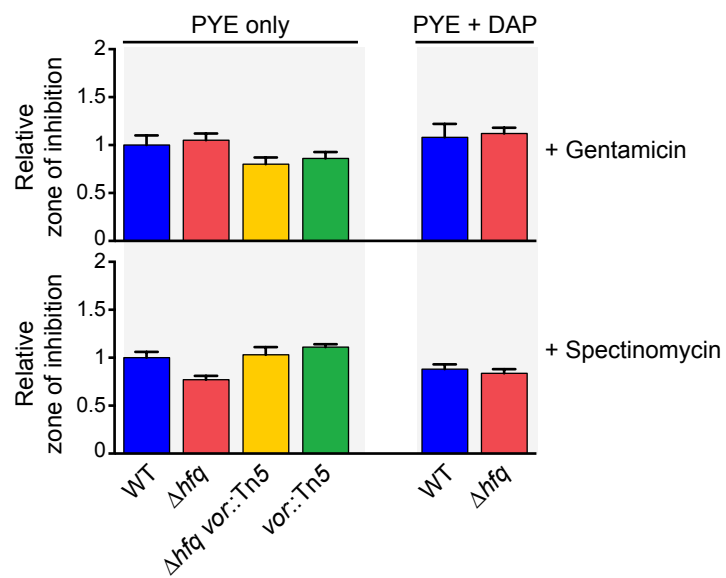

Supplement: S12 Fig — Quantification of antibiotic sensitivity for Δhfq and control strains toward gentamicin and spectinomycin. Cells from exponentially growing cultures were mixed with PYE soft agar (0.75%) and poured on top of PYE agar plates in the absence or presence of 100 μM DAP. Plates were incubated at 30°C for 70 h with antibiotic-loaded filter disks. Antibiotic sensitivity was measured as the diameter of the zone of growth inhibition around the disk relative to the zone of inhibition for the WT strain. The mean and standard deviation from 3 independent experiments are shown. (PDF) [file pgen.1006978.s012.pdf]

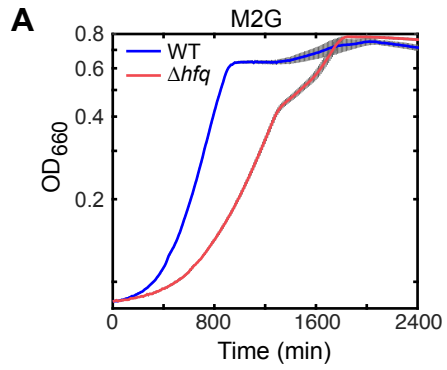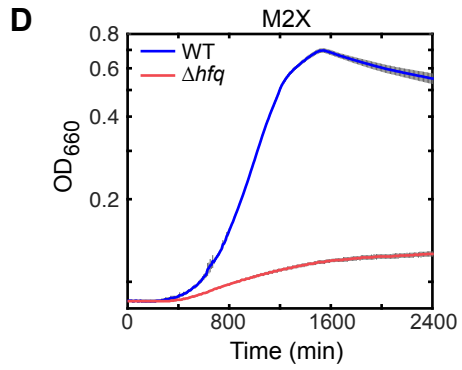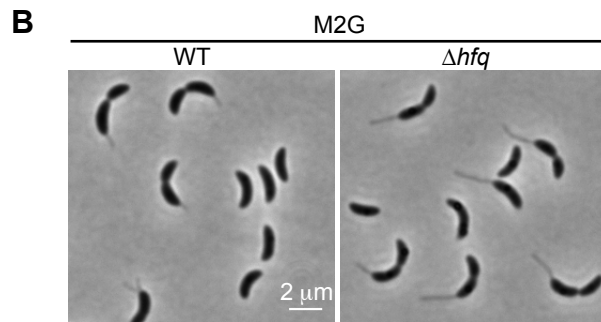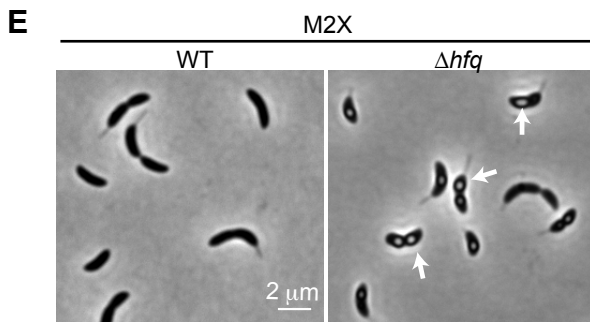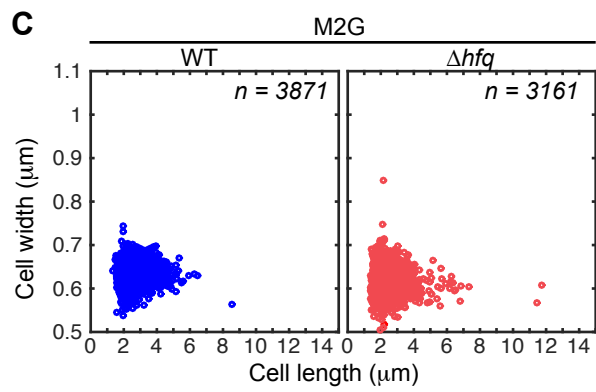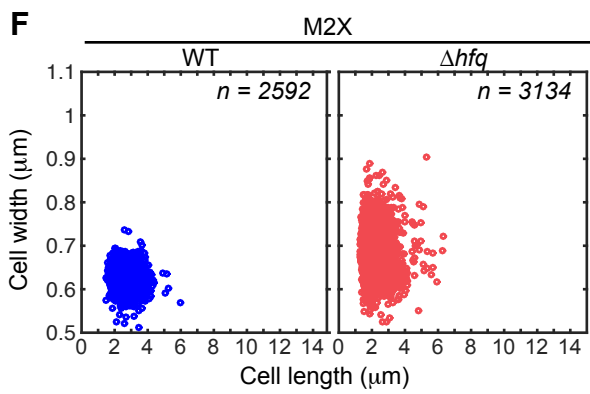

Supplement: S13 Fig — (A) Growth curves of WT and Δhfq cells at 30°C in a 96-well plate containing minimal medium with glucose as a sole carbon source (M2G). For the growth curves, each curve represents the average of 3 replicates with the standard deviation shown in grey. (B) Phase contrast images of WT and Δhfq cells grown in M2G at 30°C. (C) The measurements of cell dimensions from populations in (B). (D-F) Similar to (A-C) with cells grown in minimal medium containing xylose (M2X). Since the growth of Δhfq is severely inhibited in the presence of xylose, cells were grown in M2G before being diluted into M2X for growth measurements or imaging. Arrows denote the presence of granules in M2X-grown Δhfq cells. (PDF) [file pgen.1006978.s013.pdf]
