# Supplementary material for: Crosstalk between the tricarboxylic acid cycle and peptidoglycan synthesis in Caulobacter crescentus through the homeostatic control of α-ketoglutarate
Source: PLoS Genet. 2017 Aug 21;13(8):e1006978. doi: 10.1371/journal.pgen.1006978 (PMC5578688; doi:10.1371/journal.pgen.1006978)
Supplement: S11 Fig — Functional classification of the differentially expressed genes between WT and Δhfq strains based on COG analysis. RNA-Seq experiment was performed on cells grown in liquid PYE cultures at 30°C. About 15% of the C. crescentus genome (572 genes out of 4086) was identified as differentially expressed (fold-change ≥ 2 and p-value ≤ 0.01) between the two strains using DESeq2 from 3 biological replicates. (PDF) [file pgen.1006978.s011.pdf]

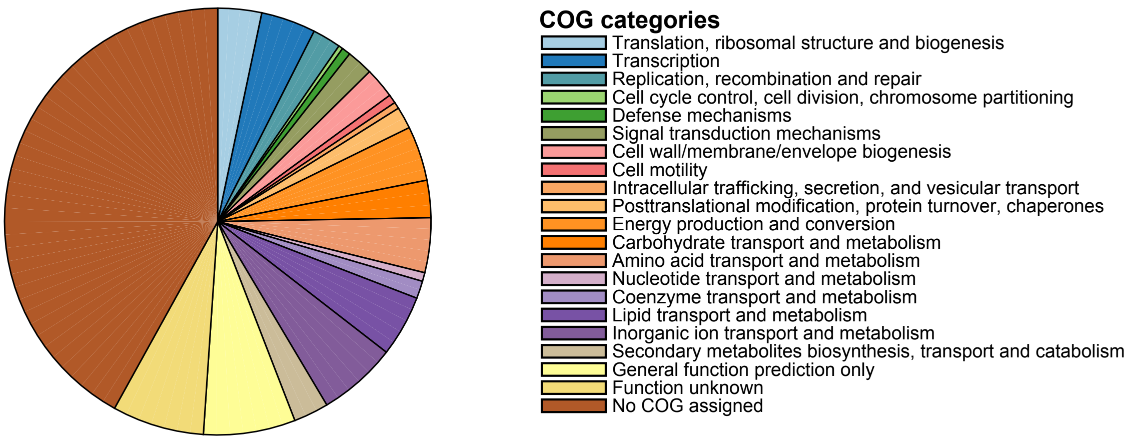
COG categories
Translation, ribosomal structure and biogenesis
Transcription
Replication, recombination and repair
Cell cycle control, cell division, chromosome partitioning
Defense mechanisms
Signal transduction mechanisms
Cell wall/membrane/envelope biogenesis
Cell motility
Intracellular trafficking, secretion, and vesicular transport
Posttranslational modification, protein turnover, chaperones
Energy production and conversion
Carbohydrate transport and metabolism
Amino acid transport and metabolism
Nucleotide transport and metabolism
Coenzyme transport and metabolism
Lipid transport and metabolism
Inorganic ion transport and metabolism
Secondary metabolites biosynthesis, transport and catabolism
General function prediction only
Function unknown
No COG assigned
