# Supplementary material for: Crosstalk between the tricarboxylic acid cycle and peptidoglycan synthesis in Caulobacter crescentus through the homeostatic control of α-ketoglutarate
Source: PLoS Genet. 2017 Aug 21;13(8):e1006978. doi: 10.1371/journal.pgen.1006978 (PMC5578688; doi:10.1371/journal.pgen.1006978)
Supplement: S5 Table — (DOCX) [file pgen.1006978.s018.docx]

**S5 Table. Strains and plasmids used in this study**

| Name | Relevant genotype or description | Reference or source |
| --- | --- | --- |
| ***C. crescentus* strains** |  |  |
| CB15N | Synchronizable variant of wild-type CB15, also named NA1000 | [1] |
| CJW5477 | CB15N Δ*hfq*::*tet* | This work |
| CJW5593 | CB15N Δ*vanA* | This work |
| CJW5632 | CB15N *vor*::Tn*5* carrying a Tn*5* insertion at nucleotide position 875 in the *vor* coding region | This work |
| CJW5633 | CB15N Δ*hfq*::*tet* *vor*::Tn*5* carrying a Tn5 insertion at nucleotide position 875 in the *vor* coding region | This work |
| CJW5893 | CB15N Δ*vanA*::pV-dCas9^hum-RBSmut1^ / psgRNA-dapE | This work |
| CJW5898 | CB15N *sucA1* (ts) carrying a V389K mutation in *sucA* (G355861 -> A355861, T355862 -> A 355862), and intergenic point mutation between *CCNA_00397* and *CCNA_00398* (T415,311 ->C415,331) | This work |
| CJW5899 | CB15N *sucA2* (ts) encodes a L707Q mutation in *sucA* (T356816 -> A356816), silent point mutations in C*CNA_01374* (T1489874 -> C1489874) and *CCNA_03449* (A3614015 -> T3614015), plus an intergenic point mutation between *CCNA_03255* and *CCNA_03256* (A3427108 -> T3427108) | This work |
| CJW5924 | CB15N Δ*hfq*::*tet* Δ*vor*::Ω Δ*vanA /* pBVMCS2 | This work |
| CJW5925 | CB15N Δ*hfq*::*tet* Δ*vor*::Ω Δ*vanA* / pBVMCS2-vor | This work |
| CJW5926 | CB15N Δ*hfq*::*tet* Δ*vor*::Ω Δ*vanA /* pBVMCS2-vorE84A | This work |
| CJW5937 | CB15N Δ*vanA*::pV-dCas9^hum^ | This work |
| CJW5938 | CB15N Δ*vanA*::pV-dCas9^hum-RBSmut1^ | This work |
| CJW5940 | CB15N Δ*vanA*::pV-dCas9^hum-RBSmut1^ / psgRNA-ftsZ | This work |
| CJW6126 | CB15N Δ*hflX*::*Ω* | This work |
| CJW6130 | CB15N Δ*vor*::Ω | This work |
| CJW6131 | CB15N Δ*hfq*::*tet* Δ*vor*::Ω | This work |
| CJW6260 | CB15N Δ*hfq*::*tet* Δ*vanA* | This work |
| CJW6265 | CB15N *vor*::pGFPC4-*vor*' | This work |
| CJW6266 | CB15N Δ*hfq*::*tet* *vor*::pGFPC4-*vor*' | This work |
| CJW6268 | CB15N Δ*hfq*::*tet* Δ*vor*::Ω Δ*vanA* | This work |
| CJW6269 | CB15N Δ*vanA*::pV-dCas9^hum-RBSmut1^ / psgRNA-sucA | This work |
| CJW6272 | CB15N Δ*vanA*::pV-dCas9^hum^ / psgRNA-ftsZ | This work |
| ***E. coli* strains** |  |  |
| DH5α | F-, *Δ(argF-lac)169*, *φ80dlacZ58(M15)*, Δ*phoA8*, *glnX44*(AS), *λ*-, *deoR481*, *rfbC1,* *gyrA96*(NalR), *recA1*, *endA1*, *thiE1*, *hsdR17*. Cloning strain | Invitrogen |
| EC100D *pir-116* | *F^-^ mcrA Δ(mrr-hsdRMS-mcrBC) φ80dlacZΔM15 ΔlacX74 recA1 endA1 araD139 Δ(ara, leu)7697 galU galK λ- rpsL (Str^R^) nupG pir-116(DHFR).* Cloning strain | Epicentre |
| JW0716-1 | F-, Δ(*araD*-*araB*)*567*, Δ*l*acZ4787(::*rrnB-3*), Δ*sucB776*::*kan*, *λ*-, *rph*-1, Δ(*rhaD-rhaB*)*568*, *hsdR514* | [2] |
| S17-1 | *recA* *pro* *hsdR* RP4-2-Tc::Mu-Km::Tn*7*, used for conjugation. Cloning strain | [3] |
| S17-1 λ*pir* | λ*pir* lysogen of S17-1. Cloning strain | [4] |
| **Plasmids** |  |  |
| pBOR | pBluescript with Ω cassette from pHP45Ω cloned at EcoRI site, spectinomycin-resistant | C. Stevens, unpublished |
| pBVMCS2 (pMT335) | Replicative, high-copy number plasmid for expression of proteins under control of vanillic acid-inducible promoter in *C. crescentus*, kanamycin-resistant | [5] |
| pBVMCS2-vor | Plasmid for expression of wild-type *vor* under control of vanillic acid-inducible promoter, kanamycin-resistant | This work |
| pBVMCS2-vorE84A | Plasmid for expression of catalytically inactive *vor* (E84A mutation) under control of vanillic acid-inducible promoter, kanamycin-resistant | This work |
| pBXMCS2 (pMT464) | Replicative, high-copy number plasmid for expression of proteins under control of xylose-inducible promoter in *C. crescentus*, kanamycin-resistant | [5] |
| pdCas9-humanized | Replicative plasmid carrying catalytically inactive Cas9 (D10A H480A, termed ‘dCas9’) which has been codon-optimized for expression in mammalian cells, ampicillin-resistant | [6] |
| pGFPC4 (pMT656) | Non-replicative plasmid in *C. crescentus*, used to create C-terminal GFP protein fusion expressed from the endogenous chromosomal locus, gentamycin-resistant | [5] |
| pGFPC4-vor’ | Integrative plasmid to create C-terminal GFP fusion to *vor* from the endogenous chromosomal locus, gentamycin-resistant | This work |
| pNPTS138 | Plasmid for gene deletion. *mobRP4^+^* *sacB* ColE1 *ori*, kanamycin-resistant | M.R. Alley, unpublished |
| pNPTS138-hflXKO-Ω | Integrative plasmid to replace *hflX* (*CCNA_01820*) with an Ω cassette, spectinomycin- and kanamycin-resistant | This work |
| pNPTS138-hfqKO-Tet | Integrative plasmid to replace *hfq* with an oxytetracycline resistance cassette, tetracycline- and kanamycin-resistant | This work |
| pNPTS138-vanAKO | Integrative plasmid to create an in-frame deletion of *vanA*, kanamycin-resistant | This work |
| pNPTS138-vorKO-Ω | Integrative plasmid to replace *vor* (*CCNA_03280*) with an Ω cassette, spectinomycin- and kanamycin-resistant | This work |
| psgRNA | Replicative plasmid carrying sgRNA targeting *mRFP* under a constitutive promoter for expression in *E. coli*, Amp^r^ | [6] |
| psgRNA-base | pBXMCS2-derivative. Plasmid carrying sgRNA with BbsI restriction sites (instead of the 20-nt targeting sequence) under a constitutive promoter. BbsI site in the original pBXMCS2 backbone was removed by site-directed mutagenesis. This was used for cloning the 20 nt gene targeting sequence, tetracycline-resistant | This work |
| psgRNA-dapE | Plasmid carrying sgRNA targeting *dapE* (*CCNA_00277*), sgRNA under a constitutive promoter, tetracycline-resistant | This work |
| psgRNA-ftsZ | Plasmid carrying *ftsZ*-targeting sgRNA under a constitutive promoter, tetracycline-resistant | This work |
| psgRNA-sucA | Plasmid carrying sgRNA targeting *sucA* (*CCNA_00342)*, sgRNAunder a constitutive promoter, tetracycline-resistant | This work |
| pVdCas9hum | pVYFPC5-derivative. Plasmid to express humanized dCas9 (‘dCas9^hum^’) under control of vanillic acid-responsive promoter from *vanA* locus, kanamycin-resistant | This work |
| pVdCas9hum-RBSmut1 | pVYFPC5-derivative. Plasmid to express humanized dCas9 (dCas9^hum^) with mutated ribosome binding site (RBS) under control of vanillic acid-responsive promoter from *vanA* locus, kanamycin-resistant | This work |
| pVYFPC5 (pMT571) | Non-replicative plasmid in *C. crescentus*, used to create C-terminal YFP protein fusion expressed from the *vanA* locus and under the control of vanillic acid-responsive promoter, tetracycline-resistant | [5] |

**References**

1. Evinger M, Agabian N. Envelope-associated nucleoid from *Caulobacter crescentus* stalked and swarmer cells. J Bacteriol. 1977;132(1):294-301. PMID: 334726

2. Baba T, Ara T, Hasegawa M, Takai Y, Okumura Y, Baba M, et al. Construction of *Escherichia coli* K-12 in-frame, single-gene knockout mutants: the Keio collection. Mol Syst Biol. 2006;2:2006 0008. doi: 10.1038/msb4100050 PMID: 16738554

3. Simon R. High frequency mobilization of gram-negative bacterial replicons by the in vitro constructed Tn*5*-Mob transposon. Mol Gen Genet. 1984;196(3):413-20. PMID: 6094969

4. de Lorenzo V, Timmis KN. Analysis and construction of stable phenotypes in gram-negative bacteria with Tn*5*- and Tn*10*-derived minitransposons. Methods Enzymol. 1994;235:386-405. PMID: 8057911

5. Thanbichler M, Iniesta AA, Shapiro L. A comprehensive set of plasmids for vanillate- and xylose-inducible gene expression in *Caulobacter crescentus*. Nucleic Acids Res. 2007;35(20):e137. doi: 10.1093/nar/gkm818 PMID: 17959646

6. Qi LS, Larson MH, Gilbert LA, Doudna JA, Weissman JS, Arkin AP, et al. Repurposing CRISPR as an RNA-guided platform for sequence-specific control of gene expression. Cell. 2013;152(5):1173-83. doi: 10.1016/j.cell.2013.02.022 PMID: 23452860
