# Supplementary material for: Crosstalk between the tricarboxylic acid cycle and peptidoglycan synthesis in Caulobacter crescentus through the homeostatic control of α-ketoglutarate
Source: PLoS Genet. 2017 Aug 21;13(8):e1006978. doi: 10.1371/journal.pgen.1006978 (PMC5578688; doi:10.1371/journal.pgen.1006978)
Supplement: S6 Table — (DOCX) [file pgen.1006978.s019.docx]

**S6 Table. Construction of strains and plasmids used in this study**

| **Name** | **Construction method** |
| --- | --- |
| **Strains**^a,b^ |  |
| CJW5477 | pNPST138-hfqKO-Tet plasmid was used to delete *hfq* following a two-step gene disruption protocol with sucrose counterselection in CB15N carrying a second copy of *hfq* at the *xylX* locus. The *hfq*::*tet* allele was then transduced into CB15N by φCR30 phage transduction to create the final strain. |
| CJW5593 | pNPST138-vanAKO plasmid was used to delete *vanA* in CB15N following a two-step gene disruption protocol with sucrose counterselection. |
| CJW5632 | *vor*::Tn*5* allele was obtained from a Tn*5* mutagenesis experiment (see Text) and transduced into CB15N by φCR30 phage transduction. |
| CJW5633 | *vor*::Tn*5* allele was obtained from a Tn*5* mutagenesis experiment (see Text) and transduced into CJW5477 by φCR30 phage transduction. |
| CJW5893 | psgRNA-dapE was transformed into the S17-1 strain and introduced into CJW5938 by conjugation. |
| CJW5898 | *sucA1* (ts) strain was obtained by UV mutagenesis |
| CJW5899 | *sucA2* (ts) strain was obtained by UV mutagenesis |
| CJW5924 | pBVMCS2 plasmid was introduced into CJW6268 by electroporation. |
| CJW5925 | pBVMCS2-vor plasmid was introduced into CJW6268 by electroporation. |
| CJW5926 | pBVMCS2-vorE84A plasmid was introduced into CJW6268 by electroporation. |
| CJW5929 | *hfq*::*tet* allele from CJW5477 was introduced into CJW5927 by φCR30 phage transduction. |
| CJW5930 | *hfq*::*tet* allele from CJW5477 was introduced into CJW5928 by φCR30 phage transduction. |
| CJW5937 | pV-dCas9^hum^ plasmid was introduced into CJW5593 by electroporation. |
| CJW5938 | pV-dCas9^hum-^RBSmut1 plasmid was introduced into CJW5593 by electroporation. |
| CJW5940 | psgRNA-ftsZ was transformed into the S17-1 strain and introduced into CJW5938 by conjugation. |
| CJW6126 | pNPST138-hflXKO-Ω plasmid was used to delete *hflX* in CB15N following a two-step gene disruption protocol with sucrose counterselection. |
| CJW6130 | pNPST138-vorKO-Ω plasmid was used to delete *vor* in CB15N following a two-step gene disruption protocol with sucrose counterselection. |
| CJW6131 | *hfq*::*tet* allele from CJW5477 was introduced into CJW6130 by φCR30 phage transduction. |
| CJW6260 | *hfq*::*tet* allele from CJW5477 was introduced into CJW5593 by φCR30 phage transduction. |
| CJW6265 | pGFPC4-*vor*' plasmid was introduced into CB15N by electroporation. |
| CJW6266 | pGFPC4-*vor*' plasmid was introduced into CJW5477 by electroporation. |
| CJW6268 | *vor*::Ω allele from CJW6130 was introduced into CJW6260 by φCR30 phage transduction. |
| CJW6269 | psgRNA-sucA was transformed into the S17-1 strain and introduced into CJW5938 by conjugation. |
| CJW6271 | *hfq*::*tet* allele from CJW5477 was introduced into CJW6252 by φCR30 phage transduction. |
| CJW6272 | psgRNA-ftsZ was transformed into the S17-1 strain and introduced into CJW5937 by conjugation. |
| **Plasmids**^c^ |  |
| pBVMCS2-vor | *vor* coding region was amplified from CB15N genomic DNA using primers irv1609/irv1623. The resulting fragment was ligated to a plasmid backbone (amplified from pBVMCS2 with primers irv1607/irv1608) using Gibson assembly, resulting in *vor* integration downstream of the vanillic acid-inducible promoter. |
| pBVMCS2-vorE84A | To create vorE84A construct, *vor* was first amplified in two halves (irv1672/irv1704 and irv1703/irv1673) with the mutations incorporated into the primers irv1704 & irv1703. The two fragments were then ligated into an intermediate plasmid digested with NdeI and KpnI using Gibson assembly. The full vorE84A construct was then amplified using primers irv1609/irv1623 and ligated into a plasmid backbone (amplified from pBVMCS2 using primers irv1607/irv1608) using Gibson assembly, resulting in *vorE84A* integration downstream of the vanillic acid-inducible promoter. |
| pGFPC4-vor’ | 3`-end of *vor* / *CCNA_03280* (~600 nt) was amplified from CB15N genomic DNA using primers irv1611/irv1612. The resulting fragment was ligated into pGFPC4 plasmid previously digested with NdeI & KpnI using Gibson assembly. |
| pNPTS138-CCNA_00123KO-Ω | ~700 bp upstream (UP) and ~700 bp downstream (DOWN) of the *CCNA_00123* coding region were amplified with primers irv1689/irv1690 and irv1691/irv1692, respectively. Ω cassette was amplified from pBOR using primers irv1647/irv1648. UP, Ω and DOWN fragments were ligated into HindIII/NheI-digested pNPTS138 by Gibson assembly. |
| pNPTS138-CCNA_02251KO | ~700 bp upstream (UP) and ~700 bp downstream (DOWN) of the *CCNA_02251* coding region were amplified with primers irv1681/irv1682 and irv1683/irv1684, respectively. UP and DOWN fragments were ligated into HindIII/NheI-digested pNPTS138 by Gibson assembly. |
| pNPTS138-CCNA_02357KO-Ω | ~700 bp upstream (UP) and ~700 bp downstream (DOWN) of the *CCNA_02357* coding region were amplified with primers irv1693/irv1694 and irv1695/irv1696, respectively. Ω cassette was amplified from pBOR using primers irv1647/irv1648. UP, Ω and DOWN fragments were ligated into HindIII/NheI-digested pNPTS138 by Gibson assembly. |
| pNPTS138-hfqKO-Tet | ~800 bp upstream (UP) and ~800 bp downstream (DOWN) of the *hfq* coding region were amplified with primers irv1337/irv1514 and irv1515/irv1340, respectively. Oxytetracycline resistance cassette (Tet) was amplified from pXCFPC5 (pMT605) [1] plasmid using primers irv1419/irv1420. UP, Tet and DOWN fragments were ligated into HindIII/NheI-digested pNPTS138 by Gibson assembly. |
| pNPTS138-vanAKO | ~600 bp upstream (UP) and ~600 bp downstream (DOWN) of the *vanA* coding region were amplified with primers irv1636/irv1637 and irv1638/irv1639, respectively. UP and DOWN fragments were ligated into HindIII/NheI-digested pNPTS138 by Gibson assembly. |
| pNPTS138-vorKO-Ω | ~700 bp upstream (UP) and ~700 bp downstream (DOWN) of the *vor* coding region were amplified with primers irv1619/irv1649 and irv1650/irv1622, respectively. Ω cassette was amplified from pBOR using primers irv1647/irv1648. UP, Ω and DOWN fragments were ligated into HindIII/NheI-digested pNPTS138 by Gibson assembly. |
| psgRNA-base | pBXMCS2 plasmid was amplified using primers irv1291/irv1702 to remove the Pxyl promoter. The resulting fragment containing the plasmid backbone was ligated with a gBlock fragment (IDT) containing sgRNA-base sequence with a constitutive promoter (J23119) using Gibson assembly. The resulting plasmid was then subjected to site-directed mutagenesis (using primers irv1708/irv1709) to remove BbsI restriction site in the backbone. The final plasmid contains only two BbsI sites in the sgRNA-base sequence for cloning purposes. |
| psgRNA-dapE | Twenty-mer guide sequence for targeting *dapE* was encoded in a pair of forward and reverse primers irv1909/irv1910. The primer pair was phosphorylated, annealed, and ligated into BbsI-digested psgRNA-base as described by Cong et al. [2] with modifications described in the Materials and Methods section. |
| psgRNA-ftsZ | Twenty-mer guide sequence for targeting *ftsZ* was encoded in a pair of forward and reverse primers irv1700/irv1722. The primer pair was phosphorylated, annealed, and ligated into BbsI-digested psgRNA-base as described by Cong et al. [2] with modifications described in the Materials and Methods section. |
| psgRNA-sucA | Twenty-mer guide sequence for targeting *sucA* was encoded in a pair of forward and reverse primers irv1840/irv1841. The primer pair was phosphorylated, annealed, and ligated into BbsI-digested psgRNA-base as described by Cong et al. [2] with modifications described in the Materials and Methods section. |
| pVdCas9hum | Humanized dCas9 (dCas9hum) was amplified from pdCas9-humanized plasmid using primers irv1699/1490. The resulting fragment was ligated into a plasmid backbone (amplified from pVYFPC5 plasmid using irv1608/irv1628 to remove *yfp* coding region) using Gibson assembly, resulting in *dCas9^hum^* integration downstream of the vanillic acid-inducible promoter. |
| pVdCas9hum-RBSmut1 | The ribosome binding site (RBS) for dCas9 in pVdCas9hum plasmid was modified from ‘GAGGAAA’ 🡪 ‘GAGGGAA’ to generate pVdCas9hum-RBSmut1. Primers irv1628/irv1729 were used to amplify plasmid backbone from pVdCas9hum plasmid with RBS mutations encoded in irv1729. The resulting fragment was ligated with dCas9 coding region (amplified from pVdCas9hum using primers irv1733/irv1490) by Gibson assembly, resulting in *dCas9^hum-RBSmut1^* integration downstream of the vanillic acid-inducible promoter. |

^a^Gene deletion using a two-step gene deletion protocol with sucrose counterselection was done as described previously [3]

^b^Phage transduction with φCR30 and conjugation using S17-1 strain were performed as described previously [4]

^c^Gibson assembly protocol was adapted from Gibson et al. [5]

**References**

1. Thanbichler M, Iniesta AA, Shapiro L. A comprehensive set of plasmids for vanillate- and xylose-inducible gene expression in *Caulobacter crescentus*. Nucleic Acids Res. 2007;35(20):e137. doi: 10.1093/nar/gkm818 PMID: 17959646

2. Cong L, Zhang F. Genome engineering using CRISPR-Cas9 system. Methods Mol Biol. 2015;1239:197-217. doi: 10.1007/978-1-4939-1862-1_10 PMID: 25408407

3. Gay P, Le Coq D, Steinmetz M, Berkelman T, Kado CI. Positive selection procedure for entrapment of insertion sequence elements in gram-negative bacteria. J Bacteriol. 1985;164(2):918-21. PMID: 2997137

4. Ely B. Genetics of *Caulobacter crescentus*. Methods Enzymol. 1991;204:372-84. PMID: 1658564

5. Gibson DG, Young L, Chuang RY, Venter JC, Hutchison CA, 3rd, Smith HO. Enzymatic assembly of DNA molecules up to several hundred kilobases. Nat Methods. 2009;6(5):343-5. doi: 10.1038/nmeth.1318 PMID: 19363495
