# Supplementary material for: Crosstalk between the tricarboxylic acid cycle and peptidoglycan synthesis in Caulobacter crescentus through the homeostatic control of α-ketoglutarate
Source: PLoS Genet. 2017 Aug 21;13(8):e1006978. doi: 10.1371/journal.pgen.1006978 (PMC5578688; doi:10.1371/journal.pgen.1006978)
Supplement: S7 Table — (DOCX) [file pgen.1006978.s020.docx]

**S7 Table. Oligonucleotides used in this study**

| **Name** | **Sequence 5` 🡪 3`** |
| --- | --- |
| irv1291 | ATATCGAATTCCTGCAGCCCGGGGGATC |
| irv1337 | GTGCAATTGAAGCCGGCTGGCGCCAAGCTTCAACAAGGTGCGTGAAGGGCTTTG |
| irv1340 | CATCCGGAGACGCGTCACGGCCGAAGCTAGCCTTCACGGTGCGAAGCGTCGGGT |
| irv1419 | CGAGGCCCTTTCGTCTTCAAG |
| irv1420 | CGAGCGCGGCCGTGAGGCTT |
| irv1490 | ATCCCCCGGGCTGCAGCTAGCTTAATCCCCTCCGAGCTGTGAGAGGTCAA |
| irv1514 | CTTGAAGACGAAAGGGCCTCGACGGGGGTTCCCCTCTCATTGTTC |
| irv1515 | AAGCCTCACGGCCGCGCTCGCCCTAGAGCCGTTTGTCCAAATCC |
| irv1607 | GTCGACGGTATCGATAAGCTTGATATCG |
| irv1608 | CATATGTTCGGTACCCATCGTCGTTTCCTCGCATCGTGGTTCGGCG |
| irv1609 | GAGGAAACGACGATGGGTACCGAACATATGCGGCACTCAGAAGTCACTCTCG |
| irv1611 | TAAGCTTTCGCGAGACGTCCAATTGCATATGGTCAAGGAAGACACGACCCCGACG |
| irv1612 | GAATTCTCCGGAGCTCGAGATCTTAAGGTACCCCCCGCCCACTGCGTCCAGAGCTTC |
| irv1619 | GTGCAATTGAAGCCGGCTGGCGCCAAGCTTCGACCCCTCATCCGTCGACTGCGTC |
| irv1622 | CATCCGGAGACGCGTCACGGCCGAAGCTAGCCCATGCCGAAACCCGAGGCCACA |
| irv1623 | CGATATCAAGCTTATCGATACCGTCGACTCACCCCGCCCACTGCGTCCAGAGC |
| irv1628 | GCTAGCTGCAGCCCGGGGGATCCACTAGTTC |
| irv1636 | GTGCAATTGAAGCCGGCTGGCGCCAAGCTTGAAGGTGTTCGTACTCGGCCGG |
| irv1637 | GGGCGCGGGACGCCACCCGAACCTTGATCAGTCGTTTCCTCGCATCGTGGTTC |
| irv1638 | TGATCAAGGTTCGGGTGGCGTCCC |
| irv1639 | CATCCGGAGACGCGTCACGGCCGAAGCTAGCGCTCCTTGCGGATCTGATCCC |
| irv1647 | CAATTCCCCTGCTCGCGCAGGCTGG |
| irv1648 | ATCAGCTTAGTAAAGCCCTCGCTAG |
| irv1649 | CCAGCCTGCGCGAGCAGGGGAATTGTTCCTCCCGGGTCTTCTCGCCC |
| irv1650 | CTAGCGAGGGCTTTACTAAGCTGATGCTCACCCTCACCCTGTTCTGC |
| irv1672 | GCCGAACCACGATGCGAGGAAACGCATATGCGGCACTCAGAAGTCACTCTCG |
| irv1673 | GTAGATCTTAAGAGCTCACCACGTGGTACCTCACCCCGCCCACTGCGTCCAGAGC |
| irv1681 | GTGCAATTGAAGCCGGCTGGCGCCAAGCTTGGTGATCGCGCGCATTGTCG |
| irv1682 | GTTGGTCATGGGGAAGGTCCTTC |
| irv1683 | GAAGGACCTTCCCCATGACCAACGAATAGAGTTTGTGTCCGGAGCC |
| irv1684 | CATCCGGAGACGCGTCACGGCCGAAGCTAGCGAGTGACGTATTTTTCCAGCAGC |
| irv1689 | GTGCAATTGAAGCCGGCTGGCGCCAAGCTTCGACCGTACCGTCGGGCTTTG |
| irv1690 | CCAGCCTGCGCGAGCAGGGGAATTGTGGACGTTTCCCTGCTAGTTCG |
| irv1691 | CTAGCGAGGGCTTTACTAAGCTGATGTAGTCTGCGCATAAAAATCCTCC |
| irv1692 | CATCCGGAGACGCGTCACGGCCGAAGCTAGCCTGGTGGTGACCGCCGGTCTC |
| irv1693 | GTGCAATTGAAGCCGGCTGGCGCCAAGCTTGATCGAGCCGCCACGACCCTAAG |
| irv1694 | CCAGCCTGCGCGAGCAGGGGAATTGCCTAACGCTGTTTTGCGCGTC |
| irv1695 | CTAGCGAGGGCTTTACTAAGCTGATCGATCATCTGTGTCATCCCGG |
| irv1696 | CATCCGGAGACGCGTCACGGCCGAAGCTAGCCTGCAGATAGGCCGCCGACTTC |
| irv1699 | GAGGAAACGACGATGGGTACCGAACATATGGACAAGAAGTATTCTATCGG |
| irv1700 | TAGTGGCGGAAAGAGAAATAGCCAT |
| irv1702 | GAGGTTTCGGGCGAGGATCAGGAC |
| irv1703 | CTTCCAGGAAGGTCTGAACGCTGATCTGGCCGCCACCGCCG |
| irv1704 | CGGCGGTGGCGGCCAGATCAGCGTTCAGACCTTCCTGGAAG |
| irv1708 | CGTGGTGGTCAGCCAAAAGACACTTTCCAAG |
| irv1709 | CTTGGAAAGTGTCTTTTGGCTGACCACCACG |
| irv1722 | AAACATGGCTATTTCTCTTTCCGCC |
| irv1729 | CTTGTCCATATGTTCGGTACCCATCGTCGTTCCCTCGCATCGTGGTTCG |
| irv1733 | CGACGATGGGTACCGAACATATGGACAAG |
| irv1771 | AGGTGATGGCTGAACTGACC |
| irv1772 | GTAGGAGACGTTGCCGTTGT |
| irv1814 | TCGTCATTCCGAACCAGAAC |
| irv1815 | CGAAGTCGAGGTTGATCAGG |
| irv1840 | TAGTGGATGCCTGCGTCGTCCGCCA |
| irv1841 | AAACTGGCGGACGACGCAGGCATCC |
| irv1909 | TAGTGGGATCGATGCTGACGGAAAC |
| irv1910 | AAACGTTTCCGTCAGCATCGATCCC |
| sgRNA-base (gBlock fragment) | GTCCTGATCCTCGCCCGAAACCTCGAATTCTAAAGATCTTTGACAGCTAGCTCAGTCCTAGGTATAATACTAGTGGGTCTTCGAGTGAAGACCTGTTTAAGAGCTATGCTGGAAACAGCATAGCAAGTTTAAATAAGGCTAGTCCGTTATCAACTTGAAAAAGTGGCACCGAGTCGGTGCTTTTTTTATATCGAATTCCTGCAGCCCGGGGGATC |
