# Supplementary material for: Crosstalk between the tricarboxylic acid cycle and peptidoglycan synthesis in Caulobacter crescentus through the homeostatic control of α-ketoglutarate
Source: PLoS Genet. 2017 Aug 21;13(8):e1006978. doi: 10.1371/journal.pgen.1006978 (PMC5578688; doi:10.1371/journal.pgen.1006978)
Supplement: S1 Text — (DOCX) [file pgen.1006978.s021.docx]

**S1 Text – CRISPRi for *Caulobacter crescentus***

**Crosstalk between the tricarboxylic acid cycle and peptidoglycan synthesis in *Caulobacter crescentus* through the homeostatic control of α-ketoglutarate**

Irnov Irnov, Zhe Wang, Nicholas D. Jannetty, Julian A. Bustamante, Kyu Y. Rhee, Christine Jacobs-Wagner

**Content**

1. **General description of the CRISPRi system for *C. crescentus***
2. **Cloning protocol for sgRNA**
3. **Sequences of sgRNA-base and sgRNAs targeting *ftsZ*, *sucA* and *dapE***
4. **References**
5. **General description of the CRISPRi system for *C. crescentus***

CRISPRi consists of two parts: a small guide RNA (sgRNA), which provides target specificity, and a catalytically inactive Cas9 (dCas9) protein [1]. The dCas9 and sgRNA complex binds to the gene of interest, leading to transcriptional interference. In our system, sgRNA is expressed under a constitutive promoter from a plasmid that can replicate in *Caulobacter crescentus*, and dCas9 is expressed under a vanillic-acid-inducible promoter at the chromosomal *vanA* locus (Fig 4A).

The sgRNA construct used here was based on the one described previously [1] with several modifications. First, we utilized an improved dCas9 binding sequence (sgRNA^F+E^) as described by Chen et al. [2]. Second, we added an extra 5`-G at the +1 position of the sgRNA construct. Since many bacteria display a preference for a purine rather than a pyrimidine at the +1 position (transcription start site) [3-5], adding 5`-G should reduce the variation in transcription efficiency between different sgRNA sequences.

For expression of dCas9 in *C. crescentus*, we used a version of *S. pyogenes* dCas9 that has been codon-optimized for expression in mammalian systems (referred to as ‘humanized dCas9’ or ‘dCas9^hum^’) [1]. The codon usage of the *dCas9^hum^* gene is more similar to the typical *C. crescentus* codon usage compared to the original *S. pyogenes* *dCas9* gene sequence. In our initial experiment to repress *ftsZ* expression, we noticed that a fraction of cells in the population showed a filamentation phenotype (consistent with FtsZ depletion) even in the absence of vanillic acid. We reasoned that this was due to a leaky expression of dCas9^hum^. To reduce this “leakiness”, we modified the original ribosome binding site (RBS) for *dCas9*^hum^ from ‘GAGGAAA’ 🡪 ‘GAGGGAA’ to create *dCas9*^hum^*^-^*^RBSmut1^, which was used for all of the CRISPRi experiments described in this study. To introduce *dCas9*^hum^*^-^*^RBSmut1^ into the chromosome, plasmid pVdCas9 hum*-*RBSmut1 was integrated into the *vanA* locus by a single-crossover recombination such that the expression of dCas9^hum-RBSmut1^ was regulated by the vanillic acid-inducible promoter, Pvan, resulting in strain CJW5938.

To repress the expression of a specific gene using CRISPRi, a plasmid containing sgRNA with a specific 20-nt targeting sequence (e.g., sgRNA-ftsZ) was introduced into CJW5938 by conjugation from an *E. coli* S17.1 strain. Transcriptional repression was elicited by inducing dCas9^hum-RBSmut1^ expression with vanillic acid (0.05-0.5 mM).

1. **Cloning protocol for sgRNA**

For ease of creating sgRNA to target various genes, we first constructed a plasmid (psgRNA-base) carrying a modified sgRNA construct (termed sgRNA-base). Instead of the 20-bp targeting sequence, the sgRNA-base contained two BbsI Type II restriction sites in a divergent configuration. Digestion with BbsI resulted in the removal of BbsI recognition sites. However, the linearized plasmid retained the necessary parts for sgRNA expression: a constitutive promoter, the dCas9 binding site (handle), and a terminator sequence. A proper sgRNA construct was then constructed by ligating a short oligo duplex (containing the 20-bp targeting sequence) into BbsI-digested psgRNA-base, as previously described [6]. In our case, the oligonucleotides contained 5` and 3’ extensions compatible for cloning into psgRNA-base, as shown below:

Forward oligo: 5’-TAGTGN_20_-3`

Reverse oligo: 5`-AAACN_20_C-3`

A schematic of the overall sgRNA cloning protocol is shown below.

**
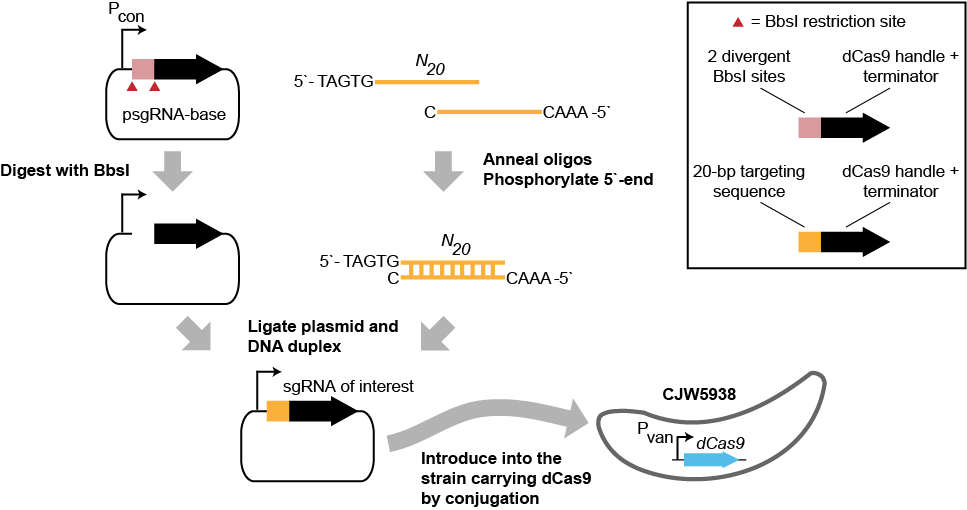
**

1. **Sequences of sgRNA-base and sgRNAs targeting *ftsZ*, *sucA*, *dapE***

**>sgRNA-base**

GAATTCTAAAGATCTTTGACAGCTAGCTCAGTCCTAGGTATAATACTAGTGGGTCTTCGAGTGAAGACCTGTTTAAGAGCTATGCTGGAAACAGCATAGCAAGTTTAAATAAGGCTAGTCCGTTATCAACTTGAAAAAGTGGCACCGAGTCGGTGCTTTTTTT

**>sgRNA-ftsZ**

GAATTCTAAAGATCTTTGACAGCTAGCTCAGTCCTAGGTATAATACTAGTGGCGGAAAGAGAAATAGCCATGTTTAAGAGCTATGCTGGAAACAGCATAGCAAGTTTAAATAAGGCTAGTCCGTTATCAACTTGAAAAAGTGGCACCGAGTCGGTGCTTTTTTT

**>sgRNA-sucA**

GAATTCTAAAGATCTTTGACAGCTAGCTCAGTCCTAGGTATAATACTAGTGGATGCCTGCGTCGTCCGCCAGTTTAAGAGCTATGCTGGAAACAGCATAGCAAGTTTAAATAAGGCTAGTCCGTTATCAACTTGAAAAAGTGGCACCGAGTCGGTGCTTTTTTT

**>sgRNA-dapE**

GAATTCTAAAGATCTTTGACAGCTAGCTCAGTCCTAGGTATAATACTAGTGGGATCGATGCTGACGGAAACGTTTAAGAGCTATGCTGGAAACAGCATAGCAAGTTTAAATAAGGCTAGTCCGTTATCAACTTGAAAAAGTGGCACCGAGTCGGTGCTTTTTTT

Legend:

in grey = constitutive promoter used previously [1]

in purple = transcription start site, +1 G

in yellow = sequence containing two divergent BbsI sites (underlined), to be substituted by 20-nt targeting sequence

in green = dCas9 binding site (handle)

in blue = transcription terminator sequence

1. **References**

1. Qi LS, Larson MH, Gilbert LA, Doudna JA, Weissman JS, Arkin AP, et al. Repurposing CRISPR as an RNA-guided platform for sequence-specific control of gene expression. Cell. 2013;152(5):1173-83. doi: 10.1016/j.cell.2013.02.022 PMID: 23452860

2. Chen B, Gilbert LA, Cimini BA, Schnitzbauer J, Zhang W, Li GW, et al. Dynamic imaging of genomic loci in living human cells by an optimized CRISPR/Cas system. Cell. 2013;155(7):1479-91. doi: 10.1016/j.cell.2013.12.001 PMID: 24360272

3. Ardissone S, Redder P, Russo G, Frandi A, Fumeaux C, Patrignani A, et al. Cell cycle constraints and environmental control of local DNA hypomethylation in alpha-proteobacteria. PLoS Genet. 2016;12(12):e1006499. doi: 10.1371/journal.pgen.1006499 PMID: 27997543

4. Kim D, Hong JS, Qiu Y, Nagarajan H, Seo JH, Cho BK, et al. Comparative analysis of regulatory elements between *Escherichia coli* and *Klebsiella pneumoniae* by genome-wide transcription start site profiling. PLoS Genet. 2012;8(8):e1002867. doi: 10.1371/journal.pgen.1002867 PMID: 22912590

5. Prados J, Linder P, Redder P. TSS-EMOTE, a refined protocol for a more complete and less biased global mapping of transcription start sites in bacterial pathogens. BMC Genomics. 2016;17(1):849. doi: 10.1186/s12864-016-3211-3 PMID: 27806702

6. Cong L, Zhang F. Genome engineering using CRISPR-Cas9 system. Methods Mol Biol. 2015;1239:197-217. doi: 10.1007/978-1-4939-1862-1_10 PMID: 25408407
